# Ion Channel Signature in Healthy Pancreas and Pancreatic Ductal Adenocarcinoma

**DOI:** 10.3389/fphar.2020.568993

**Published:** 2020-10-16

**Authors:** Julie Schnipper, Isabelle Dhennin-Duthille, Ahmed Ahidouch, Halima Ouadid-Ahidouch

**Affiliations:** ^1^Laboratory of Cellular and Molecular Physiology, UR-4667, University of Picardie Jules Verne, Amiens, France; ^2^Department of Biology, Faculty of Sciences, Ibn Zohr University, Agadir, Morocco

**Keywords:** ion channels, exocrine pancreas, pancreatic ductal adenocarcinoma, signaling pathways, biomarkers

## Abstract

Pancreatic ductal adenocarcinoma (PDAC) is the fourth most common cause of cancer-related deaths in United States and Europe. It is predicted that PDAC will become the second leading cause of cancer-related deaths during the next decades. The development of PDAC is not well understood, however, studies have shown that dysregulated exocrine pancreatic fluid secretion can contribute to pathologies of exocrine pancreas, including PDAC. The major roles of healthy exocrine pancreatic tissue are secretion of enzymes and bicarbonate rich fluid, where ion channels participate to fine-tune these biological processes. It is well known that ion channels located in the plasma membrane regulate multiple cellular functions and are involved in the communication between extracellular events and intracellular signaling pathways and can function as signal transducers themselves. Hereby, they contribute to maintain resting membrane potential, electrical signaling in excitable cells, and ion homeostasis. Despite their contribution to basic cellular processes, ion channels are also involved in the malignant transformation from a normal to a malignant phenotype. Aberrant expression and activity of ion channels have an impact on essentially all hallmarks of cancer defined as; uncontrolled proliferation, evasion of apoptosis, sustained angiogenesis and promotion of invasion and migration. Research indicates that certain ion channels are involved in the aberrant tumor growth and metastatic processes of PDAC. The purpose of this review is to summarize the important expression, localization, and function of ion channels in normal exocrine pancreatic tissue and how they are involved in PDAC progression and development. As ion channels are suggested to be potential targets of treatment they are furthermore suggested to be biomarkers of different cancers. Therefore, we describe the importance of ion channels in PDAC as markers of diagnosis and clinical factors.

## Introduction

Ion channels are plasma membrane spanning proteins found in all human tissues, allowing rapid transport of ions and fluids between the extracellular and intracellular milieu (Niemeyer et al., 2001; Gouaux and Mackinnon, 2005). Opening of ion channels can result in redistribution of different ions, which changes the electrical and chemical properties of the cell leading to several cellular processes (Roux, 2017). These include multiple signal transduction and downstream signaling events, including regulation of gene expression, secretion of enzymes and hormones, and intracellular communication between compartments (Chen et al., 1994; Tolon et al., 1996; Stock and Schwab, 2015). A stable regulation of these processes maintains normal tissue homeostasis, such as cell cycle progression, migration, and apoptosis (Kunzelmann, 2005; Kunzelmann, 2016; Prevarskaya et al., 2018; Anderson et al., 2019). Accordingly, dysregulated expression as well as altered function of ion channels are related to a great number of diseases (Kim, 2014), and can drive the transformation from normal to malignant cell behavior (Litan and Langhans, 2015). Over the past decades, aberrant and even cancer-specific expression of numerous ion channels have been demonstrated in various types of cancers (Pedersen and Stock, 2013; Djamgoz et al., 2014). Together, the abnormal expression and activity of ion channels can be categorized as “hallmarks of cancer” (Hanahan and Weinberg, 2011).

The pancreas is a complex organ, which has two main functions exerted by an exocrine and endocrine compartment (Pandiri, 2014). Dysregulation of exocrine pancreatic fluid secretion can contribute to pathologies such as pancreatitis and neoplasms such as pancreatic ductal adenocarcinoma (PDAC), whereas a well-known disorder related to dysfunction of the endocrine pancreas is diabetes mellitus (Pallagi et al., 2015; Kirkegard et al., 2017). The exocrine pancreas ensures enzymatic secretion for digesting fats and proteins in the intestines and, in parallel, the secretion of abundant fluid rich in bicarbonate ions, which serves to neutralize the acidic chime in the duodenum (Ishiguro et al., 2012; Lee et al., 2012; Pallagi et al., 2015). The bicarbonate secretion involves a tightly coordinated network of ion channels and transporters (Novak et al., 2013). The ductal epithelial cells comprising the exocrine pancreas are, as other types of epithelia, well-organized and exhibit epithelial features such as a polarized morphology and specialized cell-to-cell contact with tight junctions (Rodriguez-Boulan and Nelson, 1989). The ductal cells are equipped with a highly polarized set of ion channels and transporters, enabling the net bicarbonate excretion at the apical membrane, balanced by the net efflux of acid *via* the basolateral membrane to maintain their intracellular pH (Steward et al., 2005). Therefore, a correct distribution of ion channels and transporters is important to maintain the secreting function of exocrine pancreas (Lee et al., 2012). Moreover, expression, function, and localization of ion channels in the plasma membrane are involved in the development and progression of PDAC (Pedersen et al., 2017). PDAC can arise from ductal cells (Schneider et al., 2005) or from acinar cells transforming to ductal cells by acinar–to-ductal-metaplasia, resulting in these cells possessing a ductal phenotype (Aichler et al., 2012). The transformation-associated loss of cell polarity and cell-cell adhesions of the epithelial cell layer will result in an altered localization of ion channels (Coradini et al., 2011; Pedersen and Stock, 2013).

**Figure 1:**
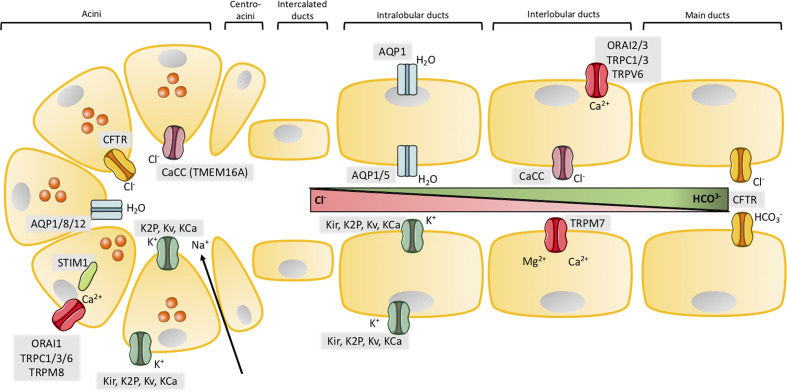
Ion channels in exocrine pancreas. Illustration of the structure of acinar and major ductal segments of secretory glands in pancreas. Acinar cells secrete digestive enzymes (orange circles in acini) and an isotonic NaCl rich fluid which transports the enzymes to the ducts. Fluid secretion in acini cells is regulated by a Cl^-^ secretion process. Cl^-^ secretion is activated by [Ca^2+^]_i_, from a Ca^2+^ influx through SOCs in the basolateral membrane, where Cl^-^ channels, Ca^2+^ activated Cl^-^ channels (CaCC) and different types of K^+^ channels are activated to provide the efflux of their respected ions. K^+^ channels also create a driving force by maintaining a negative membrane potential. The negative charge mediated by a high concentration of Cl^-^ ions results in transport of Na^+^ through tight junctions to the luminal space. NaCl makes the driving force for water to efflux through aquaporins and a cell shrinkage. This cell shrinkage reduces [Ca^2+^]_i_, which inhibits Cl^-^ and K^+^ efflux through their channels and in parallel activates basolateral transporters and pumps to restore both Cl^-^ and K^+^. The digestive enzymes are transported in the NaCl isotonic fluid to the ducts, which is low in HCO_3_^-^ concentration in the proximal ducts, but this concentration increases through the transport to the distal duct cells. The ductal fluid becomes rich in HCO_3_^-^, by a two-step process. The first step takes place in the proximal ducts, where Cl^-^/HCO_3_^-^ exchangers secretes HCO_3_^-^ and absorb Cl^-^ and Cl^-^ channels recycle Cl^-^. As in the acinar cells an osmotic reaction happens, where efflux of negative HCO_3_^-^ and Na^+^ drives water flow through aquaporins. This results in high concentration of HCO_3_^-^ (~100 mM), a low concentration of Cl^-^ (~25 mM) and a high fraction of water in the pancreatic juice. The second step takes place in the distal part of the ducts, where the specific Cl^-^ channel CFTR changes selectivity to HCO_3_^-^ and function as a HCO_3_^-^ efflux channel to determine the final concentration of the HCO_3_^-^ rich fluid (~140 mM). K^+^ channels may, as in acini, take part in the secretion of K^+^ and regulation of anion transport by maintaining the membrane potential in both the basolateral and luminal membrane. SOCs ensure the influx of Ca^2+^ which takes part in regulation of ion channels through [Ca^2+^]_i_ as in acini. Activation or inhibition of P2 receptors by Ca^2+^ signaling also regulate anion secretion through K^+^ and Cl^-^ channels.

**Figure 2:**
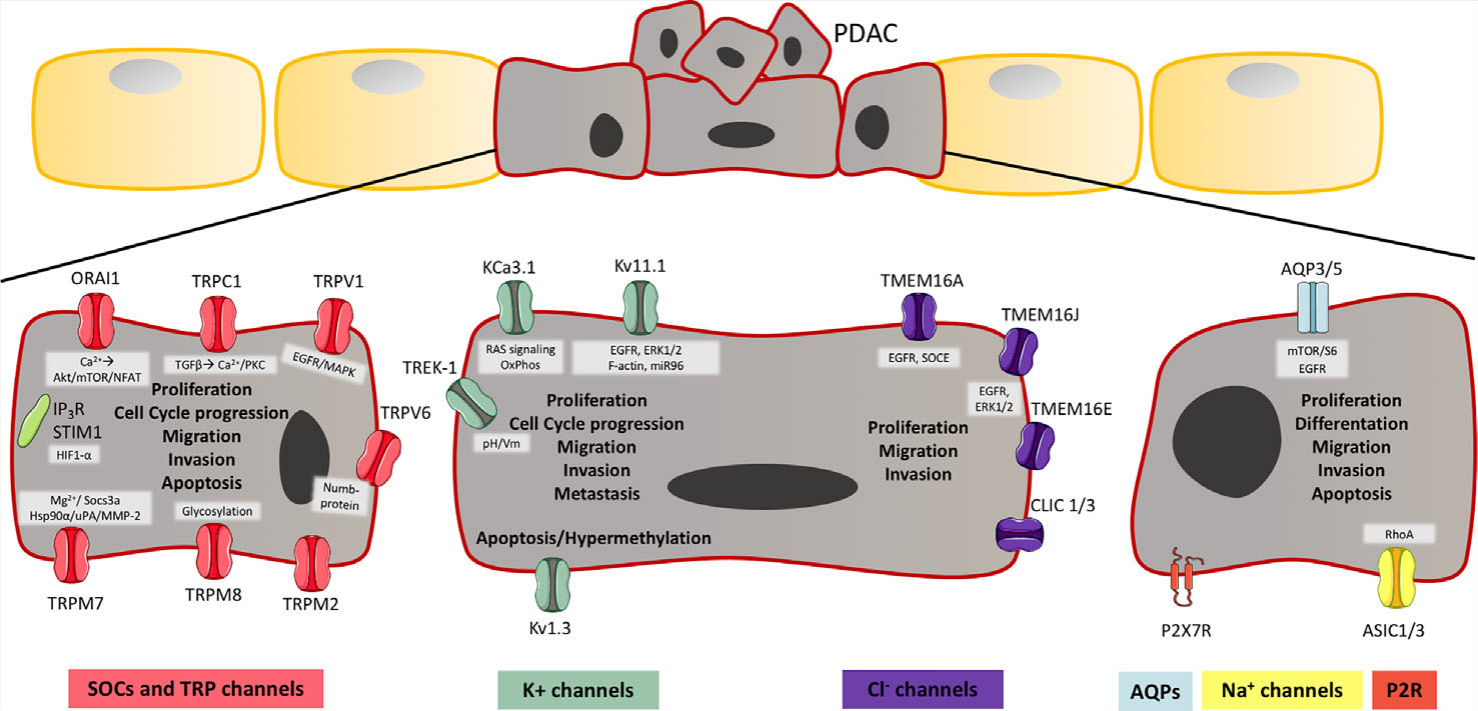
Ion channels in pancreatic ductal adenocarcinoma (PDAC). Illustration of ion channels, which have been shown to have a role in hallmarks of cancer, thereby PDAC development and progression. As cancer cells lose their polarity, the localization of the channels is unknown, and on the illustration, it should be considered that the channels have no particular localization. The aberrant expression in PDAC cells, are shown for; Store-operated channels (SOCs) and transient receptor potential (TRP) channels, K^+^ channels, Cl^-^ channels, aquaporins (AQP), Na^+^ channels and P2X7R. These channels are known to be involved in PDAC development and progression through proliferation, cell cycle progression, differentiation, migration, invasion, metastasis, and apoptosis. The known pathways and mechanism, which have been shown to be involved in these processes are shown in a grey box next to the channel and are mentioned in **Table 2**. The channels shown to be expressed in PDAC, but where the role is unknown are also shown in **Table 2**.

**Figure 3:**
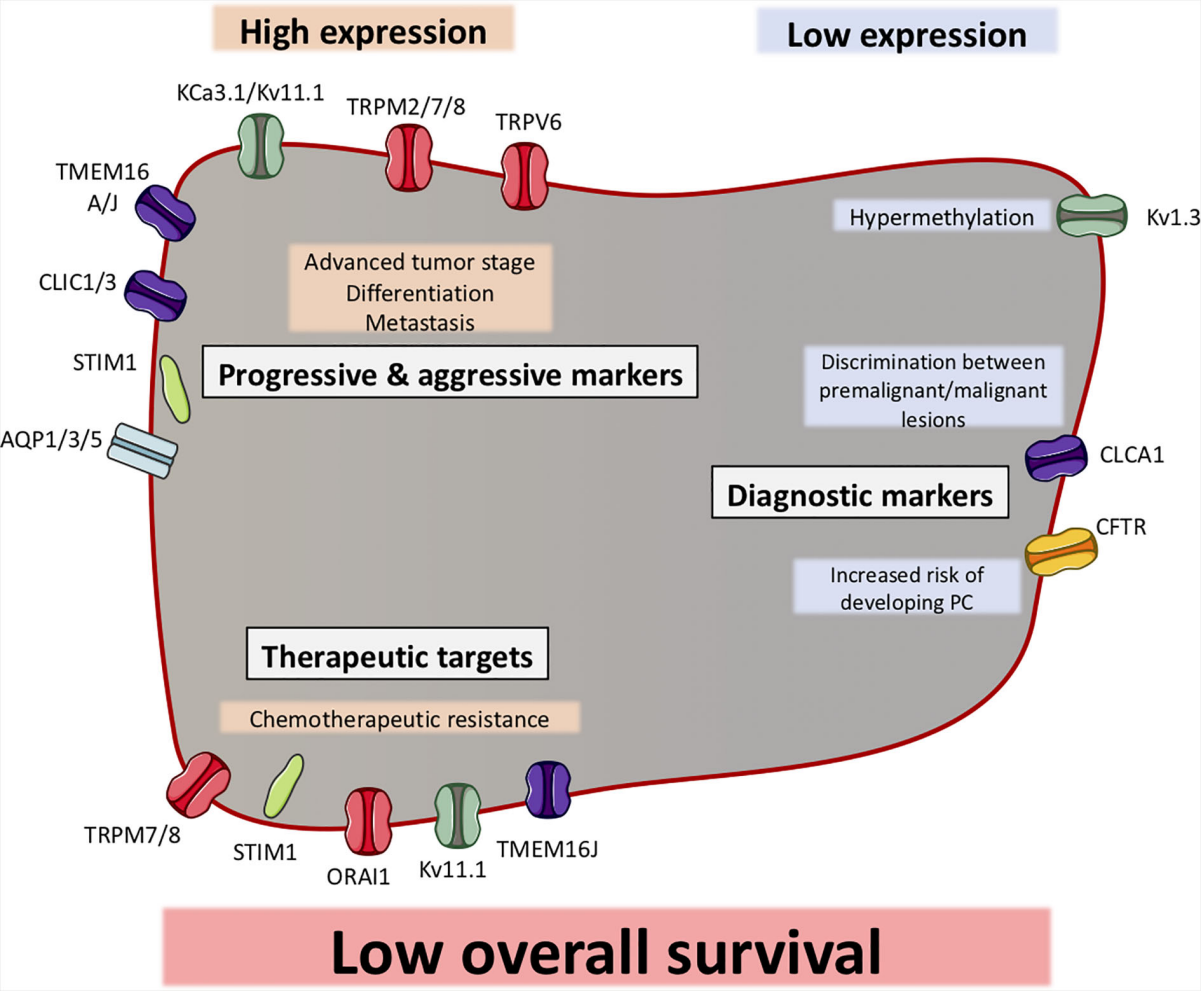
Ion channels can function as biomarkers in pancreatic ductal adenocarcinoma (PDAC). Illustration of ion channels, where the expression has been shown to be correlated with clinical factors. Most of the ion channels show a high expression in PDAC, which correlates with clinical factors (indicated in grey boxes). Some ion channels have shown to be to have a low expression in PDAC, which correlates with other clinical factors. The ion channels are grouped as progression and aggressiveness markers, diagnostic markers or therapeutic targets. Among all ion channels, their expression (except CFTR) have been shown to be correlated with a low overall survival.

Several reports and reviews about the role of transporters in bicarbonate, pancreatic fluid secretion and PDAC have been published (Novak, 2000; Lee et al., 2001; Novak et al., 2011; Ishiguro et al., 2012; Lee et al., 2012; Kong et al., 2014; Lemstrova et al., 2014; Pedersen et al., 2017; Yamaguchi et al., 2017). However, the role of ion channels in exocrine pancreas and in PDAC is not well understood. In this review, we aim to make a synthesis of the important role of ion channels and their localization and function in fluid secretion in healthy exocrine pancreatic tissue (see **Table 1** and **Figure 1**). Next, we summarize the sparse knowledge of the involvement of ion channels in PDAC progression and development *via* effects on proliferation, apoptosis, invasion and migration (see **Table 2** and **Figure 2**). Finally, we describe how ion channels are important novel biomarkers in PDAC (see **Table 2** and **Figure 3**).

**Table 1:** Expression, localization, and the potential role of ion channels in exocrine pancreas.

Channel type	Species	Pancreatic Acini	Pancreatic Duct	Localization	Function	Ref.
**K^+^ Channels**						
Kir2, Kir2.3, Kir7.1, Kir1.3	Rat	+		Basolateral	Unknown	(Kim et al., 2000) (Shuck et al., 1997)
Kir5.1 & Kir4.2	Rat Human	+	+	Unknown	Kir5.1 forms heteromeric channels with Kir4.2. Might have a role in the pH-dependent regulation of K^+^ fuxes?	(Pessia et al., 2001) (Liu et al., 2000)
TALK-1 & TALK-2	Human	+		Unknown	Highly modulated (activation) by NOS and ROS	(Duprat et al., 2005) (Girard et al., 2001)
TASK-2	Human	+	+	Luminal in duct	Might drive the force for electrogenic HCO3– secretion?	(Duprat et al., 1997) (Duprat et al., 2005) (Hayashi and Novak, 2013)
minK	Rat	+		Unknown in acinar		(Kim and Greger, 1999)
KCNQ1(KvLQT1/Kv7.) & KCNE1 (minK)	Mouse Rat	+	+	Luminal in duct Lateral and basolateral in acini	Cell volume regulation in ducts Membrane potential in acini Electrolyte/enzyme secretion with minK	(Kottgen et al., 1999) (Warth et al., 2002) (Warth and Barhanin, 2002) (Demolombe et al., 2001) (Hayashi et al., 2012)
KCa1.1 (BK, maxi-K)		+	+	Basolateral in acini/Luminal in duct	Activate luminal secretion	(Gray et al., 1990a) (Hede et al., 1999) (Hede et al., 2005)
BK (maxi-K)	Pig		+	Basolateral	Cl^-^ secretion	(Iwatsuki and Petersen, 1985a) (Iwatsuki and Petersen, 1985b) (Suzuki et al., 1985) (Petersen and Findlay, 1987) (Gallacher et al., 1984)
BK (maxi-K)	Human		+	Unknown		(Petersen et al., 1985)
BK (maxi-K)	Guinea-pig	+		Luminal	Regulation of HCO_3_^-^ secretion-induced by the bile acid Chenodeoxycholate	(Venglovecz et al., 2011)
BK (maxi-K)	Rat	+		Basolateral	Might regulate membrane potential hyperpolarzation?	(Gray et al., 1990a) (Hede et al., 2005)
IK1 (KCa3.1)	Dog (cell lines, PDEC)	+	+	Basolateral/Luminal in duct	Driving force for Cl^-^ efflux Regulate HCO_3_^-^ secretion Hyperpolarization	(Nguyen and Moody, 1998) (Thompson-Vest et al., 2006) (Jung et al., 2006)
IK1 (KCa3.1)	Mouse & Human		+	Luminal/basolateral	Setting the RMP. Involvement in anion and K^+^ transport in stimulated ducts	(Hayashi et al., 2012)
IK1 (KCa3.1)	Human & Rat					(Ishii et al., 1997) (Joiner et al., 1997) (Hede et al., 2005)
Kv3.4	Mouse	+		Unknown	Unknown	(Kalman et al., 1998)
Kv1.5	Human	+		Unknown	Might regulate membrane potential?	(Bielanska et al., 2009) (Venglovecz et al., 2015)
Kv11.1 (ERG1)	Human		+	Luminal	Might regulate membrane potential?	(Hayashi et al., 2012)
Kv10.2	Human		+	Luminal	Might regulate membrane potential?	(Hayashi et al., 2012)
**Calcium**						
ORAI1	Mouse	+		Both in acinar Mostly basolateral	Might drive exocytosis of secretory granules or stimulate fluid and electrolyte secretion?	(Lur et al., 2009) (Hong et al., 2011)
ORAI2	Dog		+	Unknown	Unknown	(Kim et al., 2013)
ORAI3	Dog		+	Basolateral	Unknown	(Kim et al., 2013)
STIM1	Mouse	+		Both in acinar Mostly basolateral	Might drive exocytosis of secretory granules or stimulate fluid and electrolyte secretion?	(Lur et al., 2009) (Hong et al., 2011)
TRPC1	Mouse	+	+	Lateral side of the basolateral membrane	Might drive exocytosis of secretory granules or stimulate fluid and electrolyte secretion?	(Hong et al., 2011)
TRPC3	Mouse	+	+	Basolateral in acini	Might drive exocytosis of secretory granules or stimulate fluid and electrolyte secretion?	(Kim et al., 2006) (Kim et al., 2009)
TRPC6	Mouse	+		Unknown	Might drive exocytosis of secretory granules or stimulate fluid and electrolyte secretion?	(Kim et al., 2006)
TRPV6	Dog		+	Unknown	Unknown	(Kim et al., 2013)
TRPM7	Zebra fish		+	Luminal	Regulating epithelial cell-cycle progression, growth, and, consequently, acinar and ductal morphogenesis, also during embryogenesis	(Yee et al., 2011)
TRPM8	Human	+	(To some extend)	Unknown	Unknown	(Yee et al., 2010)
**Chloride**						
CFTR	Rat Guinea-pig Dog Human	+	+	Luminal in ducts	Drive fluid and HCO_3_^-^ secretion	(Gray et al., 1988) (Gray et al., 1989) (Gray et al., 1990b) (Marino et al., 1991; Gray et al., 1993) (Zeng et al., 1997)
CaCC (TMEM16A)	Mouse Rat Guniea-pig Dog Bovine	+	+	Luminal on both	Drive fluid and HCO_3_^-^ secretion	(Park et al., 2001) (al-Nakkash and Cotton, 1997) (Winpenny et al., 1995) (Nguyen et al., 1997) (Yokoyama et al., 2019) (Zdebik et al., 1997) (Wang and Novak, 2013) (Park et al., 2001) (Yang et al., 2008) (Bergmann et al., 2011) (Ousingsawat et al., 2009) (Huang et al., 2009) (Randriamampita et al., 1988) (Kasai and Augustine, 1990) (Marty et al., 1984) (Gray et al., 1989) (Gray et al., 1990b)
**Sodium**						
ENaC (δ -subunit)	Mouse Human	+	+	Luminal	Unknown	(McDonald et al., 1994; Waldmann et al., 1995; Zeiher et al., 1995; Novak and Hansen, 2002; Pascua et al., 2009) (Waldmann et al., 1995)
**Other**						
AQP1	Mouse Rat Human	+	+	Both on ducts	Facilitates water flow	(Gabbi et al., 2008) (Ko et al., 2002) (Furuya et al., 2002) (Burghardt et al., 2003)
AQP5	Mouse Rat Human		+	Luminal	Facilitates water flow	(Hurley et al., 2001) (Ko et al., 2002) (Burghardt et al., 2003)
AQP8	Rat Human	+		Luminal	Facilitates water flow	(Hurley et al., 2001) (Burghardt et al., 2003) (Koyama et al., 1997)
AQP12	Mouse Chicken Human	+		Intracellular	Synthesis of digestive enzymes	(Itoh et al., 2005) (Isokpehi et al., 2009)

**Table 2:** Profile expression of ion channels in pancreatic ductal adenocarcinoma (PDAC) cell lines and tissue and how they are involved in driving PDAC formation and how channel expression correlate with clinical factors.

Channel	Profile expression Up/Downregulated (method used for expression profiling)	Cell line/Solid tumor	Driving PDAC formation in form of	Downstream regulation and signaling	Channel expression correlates with clinical factors	Ref.
**Potassium**						
KCa3.1	Upregulated (mRNA, IHC, Microarray, electrophysiological, transcriptome data, TCGA analysis)	BxPC-3 Capan-1 MiaPaCa-2 PANC-1 Solid tumors	Proliferation Cell cycle progression Migration Invasion	Ras Oxidative Phosphorylation	High expression: Low overall survival Advanced tumor stage	(Bonito et al., 2016) (Jager et al., 2004) (Hayashi et al., 2012) (Jiang et al., 2017) (Shen et al., 2017) (Kovalenko et al., 2016) (Zaccagnino et al., 2016)
KCa4.1	mRNA	Capan-1 PANC-1				(Hayashi et al., 2012)
KCa4.2	mRNA	CFPAC PANC-1				(Hayashi et al., 2012)
K_v_1.3	Downregulated in tumors (mRNA, IHC) Upregulated in cell lines (mRNA, protein)	AsPC-1 BxPC-3 Capan-1 Colo357 MiaPaCa-2 Panc-89 Panc-TUI Solid tumors	Apoptosis Hypermethylation		Low expression: Hypermethylation correlates with survival (not significant)	(Brevet et al., 2009) (Zaccagnino et al., 2017) (Bielanska et al., 2009)
K_v_1.5	Upregulated (IHC)	Solid tumors				(Bielanska et al., 2009)
K_v_7.1	Downregulated (Microarray, Nanostring electrophysiological)	A818–6 HPAF Solid tumors				(Tawfik et al., 2020) (Fong et al., 2003) (Zaccagnino et al., 2016)
minK	Downregulated (Microarray)	Solid tumors				(Zaccagnino et al., 2016)
K_v_10.1	Unknown	Solid tumors	Tumor growth	Blocking shows antitumor activity		(Gomez-Varela et al., 2007) (Pardo and Stuhmer, 2014)
K_v_11.1	Upregulated (mRNA, protein, IHC, Sequencing analysis)	BxPC-3 CFPAC-1 MiaPaCa-2 PANC-1 SW1990 T3M4 Solid tumors	Proliferation Cell cycle progression Migration Invasion Metastasis	miR96 EGFR-pathway ERK1/2 F-actin assembly	High expression: Low overall survival High Ki67 expression Advanced tumor grade	(Manoli et al., 2019) (Lastraioli et al., 2015b) (Zhou et al., 2012) (Feng et al., 2014) (Sette et al., 2013)
TREK-1	Upregulated (protein, electrophysiological)	AsPC-1 BxPC-3 Capan-1	Proliferation Migration	pH/Vm activated		(Sauter et al., 2016)
TASK-1	Downregulated (Microarray database analysis)	Solid tumors				(Williams et al., 2013)
TASK-2	(mRNA, protein electrophysiological)	HPAF				(Fong et al., 2003)
TWIK-1	Upregulated (Microarray database analysis)	Solid tumors				(Williams et al., 2013)
TWIK-3	Downregulated (Microarray)	Solid tumors	Cell differentiation			(Zaccagnino et al., 2016)
Kir3.1	Upregulated (mRNA, IHC)	Solid tumors				(Brevet et al., 2009)
Kir4.2	Downregulated (Microarray)	Solid tumors	Cell differentiation			(Zaccagnino et al., 2016)
Kir5.1	Downregulated (Microarray)	Solid tumors				(Zaccagnino et al., 2016)
**Sodium**						
VGSC SCN9A SCNA3	Downregulated (Microarray)	MiaPaCa-2 CAV Solid tumors	Proliferation	Inhibition of growth with phenytoin		(Sato et al., 1994; Koltai, 2015) (Zaccagnino et al., 2016)
ASIC1	Upregulated (mRNA, protein, IHC)	AsPC-1 BxPC-3 PANC-1 SW1990 Solid tumors	EMT Metastasis	RhoA		(Zhu et al., 2017)
ASIC3	Upregulated (mRNA, protein, IHC)	AsPC-1 BxPC-3 PANC-1 SW1990 Solid tumors	EMT Metastasis	RhoA		(Zhu et al., 2017)
**Calcium**						
ORAI1	Different expression in different cell lines (mRNA, protein) Downregulated (Microarray)	AsPC-1 BxPC-3 Capan-1 MiaPaCa-2 PANC-1 Solid tumors	Apoptosis Proliferation	Calcium-regulated Akt/mTOR/NFAT signaling		(Kondratska et al., 2014) (Khan et al., 2020) (Zaccagnino et al., 2016)
STIM1	Different expression in different cell lines Upregulated in chemo-resistant cells (mRNA, protein,TCGA analysis, IHC)	AsPC‐1 BxPC‐3 Capan-1 CFAPC‐1 MiaPaCa2 Panc‐1 Solid tumors	Apoptosis Proliferation Invasion EMT Gemcitabine resistance	Regulated by HIF1-alpha	High expression: Low disease-free survival Advanced tumor grade	(Kondratska et al., 2014) (Zhou et al., 2020) (Wang et al., 2019)
TRPM2	Upregulated (mRNA, TCGA analysis)	PANC-1 Solid tumors	Proliferation Migration Invasion		High expression: Low overall survival	(Lin et al., 2018) Reviewed in: (Stoklosa et al., 2020)
TRPM7	Upregulated (mRNA, protein, IHC, electrophysiological)	BxPC‐3 Capan-1 HPAF-II MiaPaCa2 PL45 Panc‐1 Panc 02.03 Solid tumors	Proliferation Cell cycle progression Migration Invasion	Mg^2+^-sensitive Socs3a-pathway Hsp90α/uPA/ MMP-2 proteolytic axis	High expression: Low overall survival Advanced tumor grade Advanced tumor stage Large tumor size Metastasis Molecular phenotype Treatment response	(Yee et al., 2011; Yee et al., 2012a; Yee et al., 2015) (Rybarczyk et al., 2012; Rybarczyk et al., 2017)
TRPM8	Upregulated (mRNA, protein, IHC, electrophysiological)	BxPC‐3 Capan-1 HPAF-II MiaPaCa2 Panc‐1 Panc 02.03 PL45 Solid tumors	Proliferation Cell cycle progression Apoptosis Invasion Migration	Glycosylation states	High expression: Low overall survival Low disease-free survival Poor prognosis Metastasis Molecular phenotype Treatment response	(Yee et al., 2010) (Yee et al., 2012a) (Liu et al., 2018) (Yee et al., 2014) (Du et al., 2018) (Cucu et al., 2014) (Ulareanu et al., 2017)
TRPC1	(mRNA, protein)	BxPC-1 CAPAN-1 CFPAC PANC-1	Motility	TGF-β-induced Ca/PKCα signaling		(Kim et al., 2013) (Dong et al., 2010)
TRPC4	(mRNA, protein)	BxPC-1				(Dong et al., 2010)
TRPC6	(mRNA, protein)	BxPC-1				(Dong et al., 2010)
TRPV1	Upregulated (mRNA, protein, IHC)	Capan-1 MiaPaCa-2 PANC-1 Solid tumors	Proliferation Apoptosis	EGFR/MAPK		(Huang et al., 2020) (Hartel et al., 2006) Reviewed in: (Liddle, 2007)
TRPV6	Upregulated (mRNA, protein, IHC) Downregulated (Microarray, Nanostring)	A818-6 AsPC-1 BxPC-3 CAPAN-1 CFPAC PANC-1 SW1990 Solid tumors	Cell cycle Apoptosis Metastasis	Numb protein	High expression: Low overall survival Advanced tumor stage Large tumor size Vascular infiltration	(Kim et al., 2013; Song et al., 2018) (Tawfik et al., 2020) (Zaccagnino et al., 2016)
IP3R	Immunoblotting (IF)	PANC-1	Migration	Colocalization with STIM1/ER–PM junctions		(Okeke et al., 2016)
CACNA1	Upregulated (Microarray)	Solid tumors				(Zaccagnino et al., 2016)
CACNA1G	Downregulated	Solid tumors				(Zaccagnino et al., 2016)
**Chloride**						
CLCA1	Upregulated (IHC, Proteomics)	Solid tumors	Unclear		Low expression correlates with poor prognosis Low overall survival	(Hu et al., 2018a) (Hu et al., 2018b; Hu et al., 2019)
CLCNKB	Downregulated (Microarray)	Solid tumors				(Zaccagnino et al., 2016)
CLCN1	Downregulated (Microarray)	Solid tumors				(Zaccagnino et al., 2016)
CLIC1	Upregulated (IHC)	MiaPaCa-2 PANC-1 Solid tumors	Proliferation Invasion		High expression: Low overall survival Advanced tumor grade Advanced tumor stage Large tumor size	(Jia et al., 2016) (Lu et al., 2015)
CLIC2	mRNA, protein, electrophysiological	HPAF				(Fong et al., 2003)
CLIC3	Upregulated (mRNA, protein, electrophysiological, immunohistochemistry)	HPAF Solid tumors	Promote integrin recycling		High expression: Low overall survival	(Dozynkiewicz et al., 2012) (Fong et al., 2003)
CLIC4	Upregulated (IHC)	Solid tumors	Invasion			(Zou et al., 2016)
CLIC5	(mRNA, protein electrophysiological) Downregulated (Microarray)	HPAF Solid tumors	Cell differentiation			(Fong et al., 2003) (Zaccagnino et al., 2016)
TMEM16A	Upregulated (mRNA, electrophysilogical, TCGA analysis)	AsPC-1 BxPC-3 Capan-1 MiaPaCa-2 PANC-1 Solid tumors	Migration	TMEM16A-dependent store-operated calcium entry (SOCE). EGFR-signaling pathways	High expression: Low overall survival	(Sauter et al., 2015) (Wang and Novak, 2013) (Crottes et al., 2019)
TMEM16E	Upregulated (mRNA, protein, IHC)	BxPC-3 HPAC PANC-1 Solid tumors	Proliferation Migration			(Song et al., 2019)
TMEM16J	Upregulated (mRNA, protein, IHC)	AsPC-1 BxPC-3 Capan-2 PANC-1 Solid tumors	Proliferation	ERK1/2 EGFR	High expression: Low overall survival	(Jun et al., 2017)
CFTR	Downregulated (mRNA, Microarray, Sequencing analysis) Immunoblotting (IF)	AsPC-1 BxPC-3 Capan-1 Capan-2 Colo357 CFPAC1 HPAC HPAF HS766T MiaPaCa-2 PANC-1 QGP1 S2CP9 Suit2 SW1990 T3M4 Solid tumors Organoids	EMT	Regulate expression of MUC4	*CFTR* mutation leads to a higher risk of getting pancreatic cancer	(Chambers and Harris, 1993) (Singh et al., 2007) (McWilliams et al., 2010; Cazacu et al., 2018) (Hennig et al., 2019) (Zaccagnino et al., 2016)
**Aquaporins**						
AQP1	Upregulated (mRNA, protein, IHC)	Solid tumors			High expression: Low overall survival Advanced tumor stage Large tumor size Lymph node metastasis Tumor differentiation Invasion	(Burghardt et al., 2003) (Zou et al., 2019)
AQP3	Upregulated (mRNA, Microarray, protein, IHC)	BxPC-3 Capan-2 HPAC HPAFII Solid tumors	Proliferation Apoptosis EMT	mTOR/S6 signaling Simultaneous overexpression of EGFR, Ki-67, and CK7, down-regulation of E-cadherin and vimentin	High expression: Low overall survival Advanced tumor stage Large tumor size Lymph node metastasis Tumor differentiation Invasion	(Burghardt et al., 2003) (Huang et al., 2017) (Zou et al., 2019) (Direito et al., 2017) (Zaccagnino et al., 2016)
AQP4	(mRNA)	Capan-1 Capan-2 Solid tumors				(Burghardt et al., 2003)
AQP5	Upregulated (mRNA, IHC)	Capan-1 Capan-2 HPAF Solid tumors	Proliferation Differentiation EMT	Simultaneous overexpression of EGFR, Ki-67, and CK7. Downregulation of E-cadhering and vimentin.	High expression: Low overall survival Tumor differentiation	(Burghardt et al., 2003) (Direito et al., 2017)
AQP8	mRNA Downregulated (Microarray)	Solid tumors				(Burghardt et al., 2003) (Zaccagnino et al., 2016)
**Ionotropic receptors**						
P2X4	mRNA	AsPC-1 BxPC-3 Capan-1 CFPAC-1 MiaPaCa-2 PANC-1				(Kunzli et al., 2007) (Hansen et al., 2008) (Giannuzzo et al., 2015)
P2X6	mRNA	AsPC-1 BxPC-3 Capan-1 CFPAC-1 MiaPaCa-2 PANC-1				(Kunzli et al., 2007) (Hansen et al., 2008) (Giannuzzo et al., 2015)
P2X7	Upregulated (mRNA, protein, IHC)	AsPC-1 BxPC-3 Capan-1 CFPAC-1 MiaPaCa-2 PANC-1 Solid tumors	Proliferation Apoptosis Invasion Migration	PKC, PLD ERK1/2, and JNK Decreased nitric oxide synthase		(Giannuzzo et al., 2015) (Kunzli et al., 2007) (Hansen et al., 2008) (Choi et al., 2018)
NMDAR	(IHC, Microarray)	BxPC-3 HPAFII SUIT2 Solid tumors	Proliferation Survival			(Li and Hanahan, 2013) (North et al., 2017)

## Expression, Localization, and Role of Ion Channels in Healthy Pancreatic Epithelial Cells

### Potassium Channels

The relevance of K^+^ channels in the exocrine pancreas received great attention in the 1970s–1980s, notably by Petersen’s team, thanks to electrophysiological studies of ion channels on acinar pancreatic epithelial cells dissociated from the pancreas of different animal species. While several studies have shown the expression of different families of K^+^ channels on both acinar and duct pancreatic cells (Petersen and Findlay, 1987; Bleich and Warth, 2000; Thevenod, 2002; Hayashi and Novak, 2013; Venglovecz et al., 2015), few studies have shown their physiological role in exocrine secretion.

Two excellent reviews have summarized the role of these channels in physiological process of ductal fluid secretion, likely by contributing to maintain the membrane potential and thereby providing driving forces for anion transport (Hayashi and Novak, 2013; Venglovecz et al., 2015). Among these channels the voltage- and Ca^2+^-activated K^+^, big conductance (BK, maxi-K), and the intermediate (IK, KCa3.1) Ca^2+^-activated K^+^ channels have been intensely studied in pancreatic ductal cells. The BK which is activated by cAMP and PKA is found on the basolateral membrane of rat pancreatic duct cells (Gray et al., 1990a). The authors suggest its role in pancreatic bicarbonate secretion. BK is also found mainly expressed in the apical membrane of guinea-pig non-transformed pancreatic duct epithelial cells (PDEC) (Venglovecz et al., 2011) where it regulates the bicarbonate secretion stimulated by the bile acid chenodeoxycholate likely through changes of the membrane potential. KCa3.1 was first characterized in cultured PDEC where it is expressed on the basolateral membrane of duct epithelial cells (Nguyen and Moody, 1998). Activation of P2Y2R induced an increase of free intracellular calcium ([Ca^2+^]_i_) that activates KCa3.1, which in turn hyperpolarized the membrane potential, leading to a Cl^–^dependent bicarbonate secretion (Jung et al., 2006). KCa3.1 was also found located on the basolateral and luminal membrane of pancreatic mouse and human duct cells (Hayashi et al., 2012). The same authors demonstrated that both luminal and basolateral KCa3.1 channels were involved in the regulation of membrane potential.

In acinar cells, the membrane potential created by K^+^ channels, and waves of [Ca^2+^]_i_ provide the necessary driving force for Cl^-^ efflux through the luminal membrane, which is a key step in initiating fluid and electrolyte secretion (Lee et al., 2012). The activation of K^+^ channels located on the basolateral membrane hyperpolarizes the resting membrane potential, promoting the driving force for luminal Cl^-^ efflux through Cl^-^ channels (Petersen, 2005). A Ca^2+^-dependent maxi- K^+^ channel (200 pS) has been characterized upon stimulation with acetylcholine (ACh), cholecystokinin (CCK), and bombesin in pancreatic acinar cells (Maruyama et al., 1983; Iwatsuki and Petersen, 1985a; Iwatsuki and Petersen, 1985b; Petersen et al., 1985; Suzuki et al., 1985; Petersen and Findlay, 1987). Moreover, Pearson et al. (1984) showed, on isolated pancreas acinar pig cells, that neural and hormonal (ACh, bombesin and pentagastrin) stimulation evokes a Ca^2+^-dependent cell hyperpolarization by causing an increase in membrane K^+^ conductance (Pearson et al., 1984). An intermediate Ca^2+^-activated K^+^ channel is also expressed in both the basolateral and the apical membranes of acinar cells (Thompson-Vest et al., 2006), but its role has not been studied.

KCNQ1 (KVLQT1, Kv7.1) and KCNE1 (IsK, minK) have been found in abundance in pancreatic acinar cells (Kottgen et al., 1999; Bleich and Warth, 2000; Demolombe et al., 2001; Warth and Barhanin, 2002; Warth et al., 2002; Hayashi et al., 2012). By using mouse models associated with electrophysiological studies, Warth et al. (2002) showed that KCNQ1 was predominantly located at the basolateral membrane and its co-assemblage with KCNE1 leads to a voltage-dependent K^+^ current that was increased by cholinergic stimulation and inhibited by the KCNQ1 blocker (Kim and Greger, 1999; Kottgen et al., 1999; Warth et al., 2002). The fact that inhibition of KCNQ1 channels diminishes intestinal Cl^-^ secretion, made the authors suggest its involvement in pancreatic electrolyte secretion process.

K^+^ inwardly rectifying channels (Kir) channels are expressed in exocrine pancreas. Kir 2.1, Kir2.3, Kir7.1, Kir5.1, and Kir4.2 were detected in rat pancreatic acini (Kim et al., 2000; Pessia et al., 2001). *In-situ* hybridization analysis confirmed the expression of Kir5.1 in human pancreatic acinar and ductal cells (Liu et al., 2000). Moreover, it has been suggested that Kir5.1 forms heteromeric channels with Kir4.2 in rat pancreas and is involved in the pH-dependent regulation of K^+^ flux (Pessia et al., 2001). Kir1.3 was also detected by northern blot analysis, in human pancreas (Shuck et al., 1997). The 2-Pore K^+^ channel (K_2_P) family has also been found in human exocrine pancreas; however, their localization and function are still unknown. For example, TALK-1 and TALK-2 are very specifically expressed in exocrine pancreas where they are activated by NOS and ROS (Girard et al., 2001; Duprat et al., 2005), while TASK-2 is expressed in both exocrine and endocrine pancreas (Duprat et al., 1997; Duprat et al., 2005).

### Calcium Channels

As Petersen and co-workers showed the relevance of K^+^ channels in exocrine pancreas, they have also described the role of Ca^2+^ signaling, in pancreatic acinar cells (Petersen, 2014). In the early 70’s they showed that movements of Ca^2+^ was evoked upon ACh stimulation released Ca^2+^ from intracellular stores and that only a small part of Ca^2+^ was taken up from the extracellular solution (Case and Clausen, 1973; Matthews et al., 1973). This Ca^2+^ signaling is involved in exocrine pancreatic fluid secretion as both acinar and duct cells in pancreas are regulated by receptors that change [Ca^2+^]_i_, which activates epithelial Ca^2+^-dependent K^+^ and Cl^-^ ion channels, thereby enzyme and fluid secretion (Petersen, 2014). The Ca^2+^ signal is initiated by ACh or CCK, binding to specific receptors (Case and Clausen, 1973; Matthews et al., 1973; Petersen and Ueda, 1976), which generates specific Ca^2+^ signals. These signals start by Ca^2+^ activating phospholipase C, which hydrolyzes PIP_2_, hence generating IP_3_ and diacylglycerol. IP_3_ binds to IP_3_ receptors located in the ER at the apical pole of the acinar cells mediating a Ca^2+^ wave to the basal pole (Mogami et al., 1997; Hong et al., 2011). This evokes a Ca^2+^ ER store depletion that results in clustering of the ER Ca^2+^ sensor STIM1, which activates store-operated channels (SOCs) and transient receptor potential (TRP) channels, leading to Ca^2+^ influx (Petersen and Tepikin, 2008). Members and regulators of SOCs are the SOC channel pore-forming ORAI proteins (ORAI1-3) and their regulators STIM (STIM1-2) (Hoth and Niemeyer, 2013). ORAI1 is the best described among these and are found to be expressed at the apical membrane of pancreatic acinar cells where it colocalizes with IP_3_R (Hong et al., 2011; Lur et al., 2011) and at the basolateral membrane where it colocalizes with STIM1 (Lur et al., 2011). Recently, it has been shown that inhibition of ORAI1 in pancreatic acinar cells abolished SOC entry upon stimulation with thapsigargin, CCK, and the bile acid taurolithocholic acid 3-sulfate, indicating that ORAI1 mediates SOC entry in pancreatic acinar cells (Gerasimenko et al., 2013; Wen et al., 2015).

TRPC channels have also been found to participate or influence store-dependent Ca^2+^ influx in pancreatic acinar cells. TRPC1 was found to localize both at the apical and lateral regions of the basolateral membrane, and pancreatic acinar cells isolated from TRPC1^-^/^-^ mice showed reduced Ca^2+^ influx and Ca^2+^ oscillation frequency (Hong et al., 2011). The role of TRPC1 in pancreatic acinar cells is not yet known, but it is suggested to have a similar role as in salivary glands, where they regulate fluid secretion and Ca^2+^ activated K^+^ channels (Liu et al., 2007). TRPC3 was found in the junctional site of the apical pole and the basolateral membrane of pancreatic acini cells and in TRPC3^-^/^-^ mice a reduction of Ca^2+^ influx was seen (Kim et al., 2006). Furthermore, TRPC6 seemed to be expressed in the pancreatic acini cells, but its localization and role are unknown (Kim et al., 2006). These data suggest that TRPC channels are involved in the SOC entry of pancreatic acini cells and could contribute to fluid secretion. Other TRP channels have been found to be expressed in exocrine pancreas; TRPV6 (Kim et al., 2013), TRPM7 (Yee et al., 2011) and TRPM8 (Yee et al., 2010). However, only the role of TRPM7 is described. In a zebra fish model, it has been found that TRPM7 is involved in the developmental processes of exocrine pancreas, which was linked to Mg^2+^ signaling (Yee et al., 2011). Diminish of cell cycle progression and cell growth in TRPM7-mutated zebra fish models attenuated proliferation of exocrine pancreatic epithelia. This was partially rescued by adding extra Mg^2+^ to the embryo medium (Yee et al., 2011). Furthermore, the proliferation was also regulated by suppressor of cytokine signaling 3a (socs3a), indicating that TRPM7 plays a role in the development of exocrine pancreas (Yee et al., 2011), but the physiological role in fluid secretion is yet to be determined.

In duct cells, HCO_3_^-^ secretion is mediated by cAMP/Ca^2+^ signaling systems. Through specific Ca^2+^ channels and Ca^2+^ activated ion channels (Ca^2+^-activated K^+^ and Cl^-^ channels), Ca^2+^ can act as key player in regulation and secretion of pancreatic juices (Lee M. G. et al., 2012). The localization of SOCs in duct cells, due to HCO_3_^-^ fluid secretion, is not well studied. However, it has been found that SOC-mediated Ca^2+^ influx can be a driving force for exocytosis, evoked by trypsin (Kim et al., 2008) in dog PDEC. The same authors have shown the function of SOCs in dog PDEC where the typical inward rectifying current was found, as for other types of epithelial cells (Kim et al., 2013). Furthermore, it was found that STIM1, STIM2, ORAI1, ORAI2, and ORAI3 as well as TRPC1 and TRPV6 are all expressed in dog PDEC, where ORAI3 was shown to be the dominant expressing type (Kim et al., 2013). Moreover, STIM1 and ORAI3 are colocalized in both single cell PDEC and polarized monolayers upon thapsigargin treatment (Kim et al., 2013). Using thapsigargin, the same authors showed an increased [Ca^2+^]_i_ only at the basolateral membrane, indicating that SOCs are mainly located at this site of the plasma membrane (Kim et al., 2013). It might be hypothesized that the localization of SOCs and Ca^2+^-activated ion channels are the same in pancreatic duct cells as in acinar cells, and that they play a role in HCO_3_^-^ secretion, as they play a role in enzyme and fluid secretion in acinar cells (Maleth and Hegyi, 2014).

### Aquaporin Channels

Aquaporins (AQPs) are activated by Ca^2+^ and mediate a water flow through the luminal membrane. The role of some AQP types in physiological and pathophysiological processes of exocrine pancreas has already been reviewed (Burghardt et al., 2006; Delporte, 2014; Arsenijevic et al., 2019). AQP1 is expressed at the apical and basolateral membrane of centro-acinar cells and intercalated ductal cells (Burghardt et al., 2003; Burghardt et al., 2006) and is also expressed in capillary endothelial cells and at the pancreatic zymogen granule membrane (Cho et al., 2002; Burghardt et al., 2003). AQP5 has been found to be co-localized with AQP1 in the apical membrane of centro-acinar cells and intercalated ductal cells (Burghardt et al., 2003). Otherwise, AQP8 is expressed only in acinar cells in the apical membrane (Isokpehi et al., 2009). In the two-step process of pancreatic fluid secretion, AQP8 in the pancreatic acinar cells ensures the water flow across the plasma membrane, where NaCl makes the driving force. In the pancreatic ductal cells the driving force is maintained by HCO3^-^ and Na^+^ through AQP1 and AQP5 (Burghardt et al., 2006). However, this theory is not well explained, since pancreatic fluid secretion was not found to be altered in AQP1, AQP5, AQP8, or AQP12 knockout mice (Ma et al., 2001; Yang et al., 2005; Ohta et al., 2009). Recently, the role of AQP1 has been confirmed to be involved in pancreatic fluid and bicarbonate secretion in an AQP1-knockout mouse model (Venglovecz et al., 2018).

### Chloride Channels

In the early 80’s the evidence for Ca^2+^ activated Cl^-^ channels (CaCC) were presented by whole-cell patch clamp and single-channel currents in rat lacrimal acinar. Marty and co-workers showed that Cl^-^ currents were evoked by muscarinic receptor activation and Ca^2+^, as previously demonstrated for the K^+^ current (Marty et al., 1984; Petersen, 1992). Shortly after, following investigations confirmed this Ca^2+^ activated Cl^-^ current in rat pancreatic acinar cells (Randriamampita et al., 1988). The localization of these Ca^2+^-dependent channels was proposed to be both on the basolateral and the luminal site, but speculations and further studies revealed that the localization of CaCC was found in the luminal membrane of pancreatic acinar cells, where an early activation of Cl^-^ currents was seen upon ACh stimulation (Kasai and Augustine, 1990; Zdebik et al., 1997). A small delayed current was found after Ca^2+^ has spread to the basal pole of the cell, suggesting that CaCC are highly located at the luminal membrane and to some extent in the basolateral of pancreatic acinar cells (Kasai and Augustine, 1990). New evidence shows clearly that CaCC are exclusively localized to the apical membrane and regulate pancreatic fluid secretion (Marty et al., 1984; Kasai and Augustine, 1990; Park et al., 2001).

Gray and his team have investigated the properties and roles of Cl^-^ channels in pancreatic duct epithelial cells. They and others, found two types of Cl^-^ channels in pancreatic ducts cells; cystic fibrosis transmembrane conductance regulator (CFTR), regulated by rises in [cAMP]_I_ and CaCC, regulated by an increase in [Ca^2+^]_i_ (Gray et al., 1989; Riordan et al., 1989; Gray et al., 1990b; Gray et al., 1993; al-Nakkash and Cotton, 1997; Nguyen et al., 1997). Both types of channels have been found in several species and to be localized in the apical membrane of duct cells (Gray et al., 1990b; Marino et al., 1991; Ashton et al., 1993; Evans et al., 1996; al-Nakkash and Cotton, 1997; Zeng et al., 1997; Ishiguro et al., 2002; Wang and Novak, 2013; Yokoyama et al., 2019). CaCC have been found in rodent pancreatic ducts. Here, it was shown that increases in [Ca^2+^]i, evoked by either ionomycin or ACh activated the Cl^-^ channels (Gray et al., 1990b; Gray et al., 1995). Furthermore, Cl^-^ currents were detected in mouse pancreatic ducts with no detectable function of CFTR, which indicates that these currents are carried by an ion channel that is distinct from CFTR (Gray et al., 1994; Winpenny et al., 1995).

Until now, it has been shown that mammalian TMEM16 proteins have different physiological functions. TMEM16A and B are suggested to be CaCC, where both of TMEM16E and F are suggested to have scramblase and channel activities. TMEM16D, G, and J are suggested to only have a scramblase activity. Therefore, the channel nature of all TMEM16 proteins is still not clearly identified [Reviewed in (Falzone et al., 2018)]. Recently, it has been suggested that TMEM16A, of the TMEM16/Anoctamin family, is the CaCC gene candidate for Cl^-^ secretion (Caputo et al., 2008; Schroeder et al., 2008; Yang et al., 2008). In rodent pancreatic acinar cells and intercalated ducts, expression of TMEM16A was found by immunostaining and RT-PCR (Huang et al., 2009; Yokoyama et al., 2019). The biophysical properties of the channel agreed with Ca^2+^-dependent Cl^-^ currents, described elsewhere (Yang et al., 2008). Another study demonstrated that Ca^2+^-dependent Cl^-^ secretion was defective in acinar cells from TMEM16A-null mice, indicating that TMEM16A has a physiological role in pancreatic fluid secretion (Ousingsawat et al., 2009).

The model of how Cl^-^ is secreted through channels in exocrine pancreas is described as a two-step process, starting by the activation by ACh or CCK, which trigger an IP_3_-mediated rise of cytosolic Ca^2+^ (Iwatsuki and Petersen, 1977; Reubi et al., 2003; Gautam et al., 2005; Wang and Cui, 2007). In response to this stimulation, the NaCl rich fluid starts to be produced (Hegyi and Petersen, 2013). At the basolateral membrane the Na^+^-K^+^-2Cl^-^ cotransporters (NKCC), Cl^-^/HCO3^-^ exchangers and Na^+^/K^+^ pumps are activated, to function together to establish the Cl^-^ uptake mechanism. The increased [Ca^2+^]_i_ enhances the Cl^-^ conductance of the luminal membrane and a K^+^ channel-mediated hyperpolarization of the basolateral membrane creates the driving force for Cl^-^ efflux to the luminal space. At the apical membrane, Cl^-^ ions pass through the Cl^-^ channels. This hormonal stimulation by ACh and CCK, leading to increased [Ca^2+^]_i,_ plays the central role in activating enzyme release and electrogenic Cl^-^ secretion (Petersen and Gallacher, 1988; Mogami et al., 1997; Giovannucci et al., 2002; Petersen, 2005). While Cl^-^ passes through the acinar cells a negative charge in the luminal space arises, which moves Na^+^ from the interstitial space to the acinar lumen *via* the paracellular pathway through leaky tight junctions, resulting in NaCl secretion. In physiological circumstances the acinar luminal Cl^-^ concentration contains 135 mM Cl and 25 mM HCO3- (Park et al., 2010). The second step in pancreatic fluid secretion occurs in the duct cells and depends on the high concentration of luminal Cl^-^ as it activates HCO_3_^-^ efflux through Cl^-^/HCO_3_^-^ exchangers, which elevates the luminal HCO_3_^-^ concentration and thereby activates CFTR functioning to secrete Cl^-^ and to some extend HCO_3_^-^ (Ishiguro et al., 2009; Wilschanski and Novak, 2013). The HCO_3_^-^ concentration in the fluid increases along the ducts, while the Cl^-^ concentration reciprocally decreases. By the time the pancreatic fluid leaves the ducts the ratio is inverse, with the HCO_3_^-^ concentration around 140 mM and the Cl^-^ concentration around 20 mM (Park et al., 2010). These specific concentrations will inhibit CFTR and Cl^-^/HCO_3_^-^ exchangers to prevent HCO_3_^-^ reabsorption (Wright et al., 2004).

### Sodium Channels

The efflux of Na^+^ through tight junctions in both the acinar and ductal cells is a part of regulating the HCO_3_^-^ rich fluid to be isotonic and to keep the cell osmolarity (Lee M. G. et al., 2012). The expression and function of Na^+^ channels in normal pancreatic tissue are controversial. Some studies have shown functional expression of amiloride sensitive epithelial sodium channels (ENaC) in interlobular ducts from mice (Zeiher et al., 1995; Pascua et al., 2009). Moreover, transcripts of different subunits of ENaC have been also detected in human pancreas (McDonald et al., 1994; Waldmann et al., 1995; Novak and Hansen, 2002). Other studies have shown no functional activity of ENaC in isolated small ducts from rats or in PDAC cell lines Capan-1 and HPAF (Novak and Hansen, 2002; Fong et al., 2003; Wang and Novak, 2013), which is in accordance with the secretory nature of pancreatic ducts.

## Expression of Ion Channels in PDAC Cells and Human Tissues, Function, and Associated Signaling Pathways in Cell Lines

### Potassium Channels in PDAC

#### Kv Channels

It is widely accepted that Kv channels participate in cancer development and progression and their expression has shown to be aberrant in several types of tumor tissue, also in PDAC (Serrano-Novillo et al., 2019; Teisseyre et al., 2019). It has been shown that Kv1.3 is expressed in different human PDAC cell lines, harboring mutation in p53 (Zaccagnino et al., 2017). The authors demonstrate that the inhibition of Kv1.3 by clofazimine, induces apoptosis *in-vitro* and reduces tumor weight *in-vivo* (Zaccagnino et al., 2017). Another study has reported a remodeling of Kv1.3 and Kv1.5 on a large cohort of human tissue samples (Bielanska et al., 2009). In fact, they showed that protein expression of Kv1.3 was lower in PDAC tissue, while Kv1.5 had a higher protein expression in PDAC tissue compared to healthy tissue (Bielanska et al., 2009; Comes et al., 2013). The low expression of Kv1.3 in PDAC can be explained by a hypermethylation of the *KCNA3* gene promoter (Brevet et al., 2009). Similar to Kv1.3 the expression of Kv7.1 has recently been shown to be down-regulated in PDAC (Zaccagnino et al., 2016; Tawfik et al., 2020). *KCNQ1* (gene coding for Kv7.1) was downregulated in PDAC tissue, compared to normal tissue. In addition, downregulation of *KCNQ1* was found in a system comparing PDAC A818–6 cells grown as a highly malignant undifferentiated monolayer (ML) or as three-dimensional (3D) single layer hollow spheres (HS). Database analysis showed that *KCNQ1* was involved in the enrichment of pancreatic secretion in normal pancreatic epithelium and HS, suggesting that a downregulation of *KCNQ1* might impair fluid secretion in PDAC and ML cells, while being maintained in normal pancreas and HS cells (Tawfik et al., 2020). Another comprehensive study has been investigating the gene-expression levels of the transportome in PDAC and normal specimens (Zaccagnino et al., 2016). The authors showed the downregulation of five different K^+^ channels, including the K^+^ voltage-gated channels; *KCNQ1* and *KCNE1*. Moreover, their results showed a downregulation of genes coding for the Kir4.2 (*KCNJ15*), Kir5.1 (*KCNJ16*), and the K_2_P channel TWIK-3 (*KCNK7*). In addition, the expression of *KCNJ15* and *KCNK7* was associated with the expression of EMT transcription factors (Zaccagnino et al., 2016). The authors also suggested that the higher expression of K^+^ channels in normal pancreatic epithelium takes part in setting the resting membrane potential, which generates the driving force of fluid and ion secretion in the pancreatic ducts (Zaccagnino et al., 2016).

Kv10.1 is another Kv channel that has been reported in pancreatic cancer. The expression of Kv10.1 in peripheral tissues is very restricted (Hemmerlein et al., 2006), including pancreatic tissue (Pardo et al., 1999). A xenograft mouse model of pancreatic cancer showed that monoclonal antibodies blocking the Kv10.1 current exerts antitumor activity (Gomez-Varela et al., 2007). Because Kv10.1 is nearly absent in normal tissue, there is a certain tumor selectivity for Kv10.1 expression, which gives rise to the possibility that Kv10.1 can be used as a targeting channel for the delivery of cytotoxic compounds (Pardo and Stuhmer, 2014). However, the expression and function of Kv10.1 in PDAC must be further investigated.

Interestingly, another Kv channel, Kv11.1 has been implicated as an oncogene in various cancers, including PDAC (Arcangeli et al., 2014; Lastraioli et al., 2015a). In contrast, to the Kv10.1 expression, Kv11.1 is ubiquitously expressed in normal human tissues including heart where it is mainly expressed (Sanguinetti et al., 1995; McDonald et al., 1997; Pond et al., 2000; Camacho, 2006; Comes et al., 2015). *KCNH2* (gene coding for Kv11.1) was identified as a gene with somatic mutations that could drive the metastatic process of PDAC (Zhou et al., 2012). Here, exome sequencing analysis showed that *KCNH2* clustered into a single network related to cancer development. To investigate the involvement of *KCNH2* in PDAC progression, the authors showed that knockdown of Kv11.1 reduced proliferation, colony formation and migration in PDAC cell lines. Immunohistochemical analysis of Kv11.1 expression showed expression in 8 out of 38 (21%) PDAC tissues, versus one out of 37 (2.7%) in normal tissues (Zhou et al., 2012). Another study further investigated the expression and role of Kv11.1 in PDAC (Feng et al., 2014). Here, immunohistochemical analysis confirmed a strong expression in PDAC tissues, with highest expression in the cytoplasm and membrane. In contrary, normal tissue showed only weak expression. The expression was confirmed in PDAC cell lines (Feng et al., 2014). Knockdown of Kv11.1 showed a significant decreased proliferation rate, higher number of cells undergoing apoptosis, cell cycle arrest in G1 phase and a reduction of migration and invasion, suggesting that Kv11.1 has a role in different aspects of PDAC progression (Feng et al., 2014). This was confirmed in a xenograft mouse model, were a knockdown of Kv11.1 in CFPAC-1 cells showed reduced tumor growth and fewer metastatic nodules, compared to tumors in mice injected with control cells (Feng et al., 2014). Furthermore, it was found that miR-96 was downregulated in tumor tissue and PDAC cells. The overexpression of miR-96 reduced cell proliferation, migration, and invasion *in-vitro* and reduced the Kv11.1 expression, tumor growth, and formation of metastasis *in-vivo* (Feng et al., 2014). This indicates that Kv11.1 could function as an oncogene in PDAC and be a potential target of miR-96 (Feng et al., 2014). Further investigations showed that Kv11.1 promotes pancreatic cancer cell migration, by modulation of F-actin organization and dynamics (Lastraioli et al., 2015b) suggesting its involvement in cancer metastasis (Arcangeli et al., 2014; Manoli et al., 2019).

#### KCa Channels/KCa3.1/IK

IK (KCa3.1) channels are the K^+^ channels most frequently studied among this family in PDAC. Even though, transcripts of KCa4.1 and KCa4.2 also have been shown in some cell lines (Hayashi et al., 2012). Investigation of the KCa3.1 mRNA expression in primary pancreatic cancer tumors show that 8 of 9 tumors (89%) contain a 6- to 66-fold higher expression, compared to normal pancreatic tissue (Jager et al., 2004). KCa3.1 is also found overexpressed in several PDAC cell lines (Jager et al., 2004). The over-expression of KCa3.1 was associated with an increased Ca^2+^-activated K^+^-current. Pharmacological inhibition (by TRAM-34, Clotrimazole) of KCa3.1 completely suppressed cell proliferation of MiaPaCa-2 and BxPC-3 cells but not PANC-1 cells (Jager et al., 2004). Moreover, application of [Ca^2+^]_o_ while inhibiting with TRAM-34 or Clotrimazole rescued the MiaPaCa-2 and BxPC-3 cell proliferation but did not affect this of PANC-1 suggesting that PANC-1 cell line grows independently of functional KCa3.1 channels (Jager et al., 2004). Bonito and co-workers have also reported the role of KCa3.1 in PDAC cell proliferation and migration (Bonito et al., 2016). They showed a significant mRNA upregulation of KCa3.1 in MiaPaCa-2 and BxPC-3, but not in Capan-1 and PANC-1 cells. In addition, Patch clamp measurements revealed a Ca^2+^-activated K^+^ current, which was reduced by TRAM-34 and clotrimazole. Interestingly, a transient gene silencing of KCa3.1 in MiaPaCa-2 cells completely abolished the Ca^2+^ current (Bonito et al., 2016). MiaPaca-2 cell proliferation was inhibited with TRAM-34 and 1% FBS, whereas no effect was found by application of TRAM-34 and 10% FBS in the culture media, as shown before (Jager et al., 2004). Silencing of KCa3.1 removed the ability of MiaPaCa-2 cells to proliferate, and attenuated their cell invasion and migration. Surprisingly treatment upon TRAM-34 or clotrimazole increased cell migration. It was hypothesized that this could be due to Ca^2+^ homeostasis, which was investigated by Ca^2+^ imaging that confirmed that TRAM-34 evoked an increase of [Ca^2+^]_i_ (Bonito et al., 2016) possibly leading to promotion of cell migration (Lotz et al., 2004). This indicates that KCa3.1 expression and function are important for cell proliferation, migration and invasion (Bonito et al., 2016). Another study has identified KCa3.1 as a regulator of oxidative phosphorylation in MiaPaCa-2 cells as silencing and inhibition of KCa3.1 determined the effect of channel dependent-oxidative phosphorylation in proliferation and ATP generation (Kovalenko et al., 2016). In addition, MiaPaCa-2 cells showed mRNA and protein levels in mitochondria, suggesting that KCa3.1 is involved in proliferation through metabolic processes (Kovalenko et al., 2016). Three other studies have identified *KCNN4* (gene coding for KCa3.1) as a gene related to PDAC as its transcripts and gene-level were upregulated compared to normal pancreatic tissue (Zaccagnino et al., 2016; Jiang et al., 2017; Shen et al., 2017). The upregulation of *KCNN4* was associated with the gene expression of different EMT transcription factors (Zaccagnino et al., 2016).

#### Two-Pore K^+^ Channels (K_2_P)

The outward conducting, pH and membrane potential activated K_2_P channels have an impact on physiological processes. They can regulate the cell volume, the membrane potential in form of being pH sensitive, modulate ion transport and Ca^2+^ homeostasis. They are involved in cancer progression due to their impact on cell growth survival and migration, as it has been shown in different types of cancer (Mu et al., 2003; Kim et al., 2004; Voloshyna et al., 2008; Alvarez-Baron et al., 2011; Lee G. W. et al., 2012; Nagy et al., 2014; Sauter et al., 2016). A broad data base analysis of K_2_P expression in different cancers revealed an aberrant expression of different K_2_P in PDAC (Williams et al., 2013). mRNA expression of *KCNK1* (gene coding for TWIK-1) was upregulated in PDAC compared to normal tissue, and *KCNK3* (gene coding for TASK-1) were downregulated (Williams et al., 2013). One study has found the functional mRNA and protein expression of *KCNK5* (gene coding for TASK-2) in PDAC cell lines HPAF, but the role in cancer progression was not further studied (Fong et al., 2003). In another study a pH sensitive K^+^ current was identified in BxPC-3 cells and was probably mediated by TREK-1 (Sauter et al., 2016). TREK-1 protein expression was shown in PDAC cell lines where it was shown that TREK-1 was involved in proliferation (Sauter et al., 2016). A similar pattern was shown in a scratch wound healing assay were activation of TREK-1 lead to decreased migration. These results indicate that TREK-1 has a potential inhibiting role in PDAC proliferation and migration (Sauter et al., 2016). Very few studies have been done on the role of K_2_P channels in PDAC. However, it can be suggested from other types of cancer that these channels can be related to cancer progression (Comes et al., 2015).

### Calcium Channels in PDAC

#### ORAI and STIM

It is well known that physiological Ca^2+^ signaling has many effects in the exocrine pancreas, and takes part in stimulating secretion of HCO_3_^-^ and other ions (Hegyi and Petersen, 2013; Maleth and Hegyi, 2014). In non-excitable cells, such as cancer cells, Ca^2+^ entry occurs mainly through SOCs (Mo and Yang, 2018) but also through transient receptor potential channels (TRP), which are selective for both Ca^2+^ and Na^+^ (Worley et al., 2007).

There is increasing evidence of dysregulated Ca^2+^ signaling in cancer. This evidence is based on the implication of SOCs and TRP in key hallmarks of cancer progression and as prognostic markers in several types of cancers (Prevarskaya et al., 2007; Shapovalov et al., 2016; Chen et al., 2019). Some members of SOCs and TRP have been studied in PDAC, even though knowledge is less pronounced compared to other types of cancer, such as breast-, cervical-, and colorectal cancer (Chen et al., 2019).

The complex of ORAI1 and STIM1 has been shown to play a role in carcinogenesis and to be involved in regulation of proliferation, migration, invasion and apoptosis in different types of cancer (Chen et al., 2019). Only two studies have been performed on PDAC showing that ORAI1 and STIM1 mediate SOC entry and that they are involved in proliferation, survival and apoptosis (Kondratska et al., 2014; Khan et al., 2020). It has been shown that both ORAI1 and STIM1 were expressed in several PDAC cell lines at mRNA and protein levels, with PANC-1 showing the highest levels of both. Knockdown of ORAI1 and STIM1 with siRNA showed a significant reduction of Ca^2+^ entry. This was confirmed in PANC-1, AsPC-1, MiaPaCa-2, and Capan-1 cells, indicating that SOC entry is mediated by ORAI1 and STIM1 in different PDAC cell lines. A recent study has revealed the involvement of Calcium Release-Activated calcium (CRAC) channel (ORAI1) in proliferation of PDAC (Khan et al., 2020). An inhibition with CRAC channel inhibitor, RP4010, showed a significant reduction of cell proliferation and colony formation in MiaPaCa-2 cells and in L3.6pl (a pancreatic adenosquamous carcinoma derivated cell line). The influx of calcium was also inhibited upon treatment with RP4010, suggesting that cell proliferation is mediated by regulation of Ca^2+^ entry through CRAC channel (Khan et al., 2020). It was proposed that cell proliferation was calcium-regulated through the AKT/mTOR signaling pathway as RP4010 inhibition decreased the mRNA levels and protein expression of phosphorylated AKT, modulated the expression of proteins important for downstream AKT/mTOR signaling. Furthermore, RP4010 or ORAI1 knockdown showed a decrease in mRNA levels and in nuclear translocation of NFAT1, suggesting that CRAC channel takes part in modulating calcium signaling associated with NFAT translocation and that PDAC proliferation is regulated through the calcium-activated AKT/mTOR/NFAT signaling (Khan et al., 2020). To test if RP4010 could enhance anticancer activity of standard used treatments gemcitabine and Nab-Paclitaxel, a combination of the three drugs were used to treat PDAC cell lines. The results showed a decrease in proliferation. A synergistic effect of certain dose combinations of RP4010 with gemcitabine/Nab-Paclitaxel was found to inhibit cell growth. In addition, this synergistic treatment downregulated the expression of NFATC1 and mTOR mRNA and NFAT1, NF-κB, and phosphorylated S6K proteins, suggesting that inhibition of cell proliferation through CRAC channel are mediated by a downregulation of mTOR, NFAT and NF-κB signaling (Khan et al., 2020). The anticancer activity and the synergistic effect of RP4010/Gemcitabine/Nab-Paclitaxel were tested *in-vivo*. In a patient-derived xenograft mouse model, it was shown that Ki-67 expression decreased with the treatment of RP4010 or by the triple combination treatment (Khan et al., 2020). The overexpression found by Kondratska and co-workers can explain increased [Ca^2+^]_i_ levels in PDAC cell lines, and that this is a mechanism for survival (Kondratska et al., 2014). In contrast, another study has found decreased gene expression levels of ORAI1 (Zaccagnino et al., 2016).

A recent study has been investigating the role of STIM1 in PDAC progression (Wang et al., 2019). shRNA knockdown of STIM1 showed decreased proliferation, invasion, and upregulation of E-cadherin protein levels and downregulation of vimentin levels, suggesting that STIM1 is involved in carcinogenesis of PDAC and in some way involved in Epithelial-Mesenchymal transition (EMT). Even though, E-cadherin levels have shown to be upregulated, in contrary to what is usually seen in cells undergoing EMT where E-cadherin decrease in favor of N-cadherin (Gheldof and Berx, 2013). Furthermore, tissue microarray analysis showed that the STIM1 expression positively correlated with HIF-1α (Wang et al., 2019). It was further shown that similar protein expression levels of STIM1 and HIF-1α were expressed in different PDAC cell lines. STIM1 and HIF-1α protein levels were also upregulated in some PDAC tumor samples compared to non-tumor samples. Knockdown of HIF-1α in PANC-1 cells revealed a significantly lower mRNA and protein expression of STIM1. The co-upregulation of both proteins and the downregulation of STIM1 upon knockdown of HIF-1α indicate that STIM1 is regulated by HIF-1α on the transcriptional level. STIM1 promoter activity was tested in PANC-1 cells upon normoxia or hypoxia, where HIF-1α binding sites, under hypoxic conditions reduced STIM1 promoter activity (Wang et al., 2019). These results indicate that HIF-1α probably regulates STIM1 transcription and that STIM1 overexpression, in a hypoxic environment, can promote PDAC progression and invasion (Wang et al., 2019).

The EMT process is stimulated upon loss of cell-cell contact and occurs in migrating cancer cells (Gheldof and Berx, 2013). It has been shown in disconnected individual PANC-1 cells that ER/Plasma membrane junctions containing STIM1, together with the IP_3_Rs, redistribute to the leading edge of focal adhesions (Okeke et al., 2016). An inhibition of IP_3_Rs and SOC entry reduced the migrating capacity of PANC-1 cells. This mechanism indicates the importance of Ca^2+^ signaling in migration through SOC entry and intracellular calcium channels (Okeke et al., 2016).

#### TRP Channels

TRP form an adaptable family of ion channel proteins where the majority are calcium permeable and show regulatory patterns that are sensitive to different environmental factors (Shapovalov et al., 2016). The role of TRP has been reported in different types of cancer (Prevarskaya et al., 2007). It has been proposed that TRPC1 can regulate PDAC cell proliferation through TGF-β signaling, as TGF-β has been shown to be one of the key modulators of EMT in mammary epithelial cells (Radisky and LaBarge, 2008). In PDAC cell line BxPC-3, TGF-β has shown to induce [Ca^2+^]_i_ increase leading to activation of the Ca^2+^-dependent protein kinase Cα (PKC-α) and its translocation to the plasma membrane. PKC-α activation by TGF-β initiates the motility and migration by inhibiting tumor suppressor PTEN (Chow et al., 2008). Further on, it has been shown that there is a high expression of TRPC1, TRPC4 and TRPC6 in BxPC-3 cells (Dong et al., 2010). Here, it was confirmed that TGF-β induces cytosolic Ca^2+^ concentrations through TRPC1, followed by a PKC-α activation, thus initiating motility and migration. This was shown by a pharmacological inhibition of SOC entry pathways with 2-APB and La^3+^, which abolishes the TGF-β induced cytosolic Ca^2+^ increase. Furthermore, blocking of PKC-α with selective PKC-α inhibitors inhibited the TGF-β mediated Ca^2+^ entry. In addition, knockdown of TRPC1 with siRNA reversed the effect of TGF-β on cell motility, although, knockdown of TRPC4 and TRPC6 did not have an effect on motility of TGF-β mediated BxPC-3 cell motility (Dong et al., 2010). These observations suggest that dysregulated Ca^2+^ entry through TRPC1 could be involved in EMT, and thereby invasion and metastasis of PDAC.

TRPV channels function as sensors in the central and peripheral nervous system where the majority is sensitive to voltage and temperature (Premkumar and Abooj, 2013). TRPV1 has shown to be related to oncogenesis and is expressed in different types of cancer (Domotor et al., 2005; Lazzeri et al., 2005; Sanchez et al., 2005; Miao et al., 2008; Morelli et al., 2014; Vercelli et al., 2014). TRPV1 can be activated by multiple pathways, which can promote pancreatic inflammation and pain, but also pancreatic cancer (Hartel et al., 2006; Huang et al., 2020). TRPV1 was shown to be upregulated at the mRNA and protein level in PDAC tissue compared to normal pancreatic tissue (Hartel et al., 2006). TRPV1 staining has been shown in both normal acini and ducts but with highest intensity in nerves of inflamed tissue surrounding the cancer. The elevated TRPV1 expression in infiltrating nerves was associated with pain in patients with PDAC. The same authors showed that inhibition of TRPV1 with resiniferatoxin induces apoptosis by targeting mitochondrial respiration and decreases cell growth in some PDAC cell lines (Hartel et al., 2006).

Recently, it has been shown that TRPV1 regulates the Epidermal Growth Factor Receptor (EGFR) in PANC-1 cell line (Huang et al., 2020). In this study, an overexpression of TRPV1 has been associated with a decrease in protein expression of EGFR in PANC-1. Vice versa, the downregulation and inhibition of TRPV1 increases the protein expression of EGFR. In addition, an overexpression of TRPV1 showed increased levels of ubiquitinated EGFR. The membranous fractions of EGFR were reduced, while the cytoplasmic were increased compared to the control (Huang et al., 2020). This indicates that TRPV1 promotes EGFR ubiquitination and thereby a downregulation of EGFR activity, resulting in EGFR cytoplasmic translocation and degradation, which was found to be mainly through the lysosomal pathway (Huang et al., 2020). Furthermore, it was shown that TRPV1 overexpression inhibited proliferation, probably through the MAPK signaling pathway. Overexpression of TRPV1 resulted in decreased mRNA levels of KRAS and AKT2 and a treatment with EGF reduced the protein expression of ERK, JNK, and CREB, suggesting that a TRPV1 overexpression decreases EGFR/MAPK dependent proliferation in PANC-1 cells (Huang et al., 2020). The two above mentioned studies show contrary results in form of how the expression of TRPV1 is related to proliferation. Hartel et al., demonstrated that inhibition of TRPV1 terminate cell growth and induced apoptosis, where Huang et al., found that an overexpression of TRPV1 leads to a reduced proliferation rate (Hartel et al., 2006; Huang et al., 2020).

Another member of the TRPV family, TRPV6, was also found to be overexpressed in the primary pancreatic cancer tissues at both protein and mRNA levels. Moreover, by immunohistochemical analysis, it was found that TRPV6 is mainly localized in the cytoplasm in both tumor and normal tissue (Song et al., 2018). *In-vitro*, the highest level of TRPV6 was found in two pancreatic cell lines, Capan-2 and SW1990. The knockdown of TRPV6, by siRNA, resulted in reduced proliferation, cell cycle arrest in G0/G1 phase, promotion of apoptosis, and suppression of cell migration and invasion (Song et al., 2018). Furthermore, the silencing of TRPV6 resulted in a significant increase of sensitivity to the chemotherapeutic reagent oxaliplatin (Song et al., 2018). In contrast to this finding, Zaccagnino et al., 2016 showed a downregulation of TRPV6 in PDAC tissue, compared to normal pancreatic epithelium. Moreover, Tawfik et al., 2020 found also a downregulation of TRPV6 expression in a PDAC cell line A818–6 grown in a highly malignant undifferentiated monolayer. The authors suggest that a lower expression of TRPV6 could contribute to an inhibited epithelial fluid secretion in PDAC (Tawfik et al., 2020).

The TRPM family is also constituted with several members, which have been found to be implicated in carcinogenesis. One of the most studied in PDAC is TRPM7, which is a particular channel having an intrinsic kinase, together with its closest homolog TRPM6 (Yee et al., 2012a). TRMP7 is ubiquitously expressed and controls cellular homeostasis of ions, especially Mg^2+^ and Ca^2+^. Interpreting that the developmental role of TRPM7 in zebrafish could be the same in humans, the role of TRPM7 has been studied in the development and progression of PDAC. Here, it has been shown that there was an overexpression of TRPM7 protein in PDAC tissue compared to normal tissue, and that TRPM7 is required for Mg^2+^-regulated proliferation. Knockdown of TRPM7 with siRNA showed that this channel is necessary to prevent cell cycle arrest in G0/G1 phases. Furthermore, the proliferation of TRPM7-deficient PDAC cells was rescued by adding Mg^2+^ to the cell culture medium (Yee et al., 2011). Another study confirmed the overexpression of TRPM7 both at mRNA and protein levels in PDAC tissue (Rybarczyk et al., 2012). Furthermore, it was shown that TRPM7 silenced BxPC-3 cells decreased [Mg^2+^]_i_, suggesting that TRPM7 takes part in regulating Mg^2+^ uptake in PDAC cells. In contrary to previous findings, these authors demonstrated that the silencing of TRPM7 had no effect on cell viability or proliferation, but a significant decrease of BxPC-3 cell migration (Rybarczyk et al., 2012). TRPM7 has also been found to be involved in cell invasion in both MiaPaCa2- and PANC-1 cells. In the two last cell lines, TRPM7 regulates constitutive cation currents, the influx and homeostasis of Mg^2+^, and cell invasion through the Hsp90α/uPA/MMP-2 proteolytic pathway (Rybarczyk et al., 2017).

Besides TRPM7, also other TRPM channels are found to be expressed in pancreatic cancer (Yee et al., 2010; Yee et al., 2014). TRPM8 is expressed in different types of adult human tissue and has also been found to be expressed in PDAC. In a panel of PDAC cell lines, mRNA TRPM8 was consistently overexpressed compared to the control cell line (H6c7) (Yee et al., 2010). This pattern has further been confirmed by immunohistochemistry in human PDAC tumors, compared to normal pancreatic tissue (Yee et al., 2010; Yee et al., 2014). Here, it was found that TRPM8 has a role in carcinogenesis in form of proliferation, migration and senescence. TRPM8 is required for proliferation by promoting cell cycle progression in PANC-1 and BxPC-3 cells, as a knockdown of TRPM8 showed a significant decrease in proliferation rate and a cell cycle arrest in G0/G1 phase (Yee et al., 2010). In another study, the knockdown of TRPM8 showed the opposite effect on proliferation. Here, the proliferation increased by 30% in PANC-1 cells and in contrary the proliferation was suppressed in HEK/M8 cells. It was found that TRPM8 is expressed in a non-glycosylated form in different PDAC cell lines, and that the channel in this form might have a protective role in PDAC (Ulareanu et al., 2017). Concerning the involvement of TRPM8 in migration and invasion, two studies show opposite results. One study demonstrated that TRPM8 also is required for cell migration, as a knockdown of the channel impaired migration of BxPC-3 and MiaPaCa-2 by 60% and 45%, respectively (Yee et al., 2014). Where another study found that it enhanced the motility of PANC-1 cells (Cucu et al., 2014).

Recently, it has been shown that a third member of the TRPM family, also plays a role in PDAC progression (Lin et al., 2018). An overexpression of TRPM2 enhanced the proliferative, migrative and invasive abilities of PANC-1 cells, compared to the control cells and the results were inversed when TRPM2 was silenced in PANC-1 cells. It should be noted, that the study does not mention the application of a proliferation inhibitor during the Scratch wound-healing assay, which investigates the migratory role of TRPM2. Therefore, one can speculate if the wound-healing could be caused by proliferation, and not migration. Nevertheless, these results suggest that TRPM2 is involved at least in cell growth and invasion (Lin et al., 2018).

#### Voltage-Dependent Calcium Channels

The expression of two voltage dependent Ca^2+^ channels have been found to be dysregulated in PDAC, namely, CaV2.1 (*CACNA1A*) and CaV3.1 (*CACNA1G*) are upregulated and downregulated, respectively (Zaccagnino et al., 2016). These sparse data indicate that voltage-dependent Ca^2+^ channels might have a role in PDAC progression.

### Chloride Channels in PDAC

#### Ca^2+^-Activated Chloride Channel (CaCC) and TMEM Proteins

Aberrant expression and dysregulated function of Cl^-^ channels have shown to be involved in carcinogenesis, especially their role in cell volume regulation has shown to be important for cancer cell migration and infiltration (Duran et al., 2010; Prevarskaya et al., 2010; Anderson et al., 2019). In Capan-1 cells, CaCC are expressed at the apical membrane, as shown for normal pancreatic acinar and ductal cells (Park et al., 2001; Wang et al., 2013; Wang and Novak, 2013).

The functional role of TMEM16A has been found to vary between different types of cancer (Ayoub et al., 2010; Liu et al., 2012; Ruiz et al., 2012; Britschgi et al., 2013). While a pro-proliferative role was found in breast and prostate cancer, the role in pancreatic cancer has been found to be contradictory. An anti-proliferative effect was found by a knockdown and overexpression strategy in CFPAC-1 cells (Ruiz et al., 2012), where another study found that inhibition with the TMEM16A specific inhibitor T16A_inh_-A01 decreased the proliferation rate in CFPAC-1 cells (Mazzone et al., 2012). Both studies lack the comparison of PDAC cell lines with a normal pancreatic epithelial control cell line. This was considered in a recent study, where the role of TMEM16A was investigated in PDAC cell lines and compared to a normal pancreatic epithelial control cell line (Sauter et al., 2015). The mRNA expression of TMEM16A was upregulated, with a 1,450-fold, in AsPC-1, BxPC-3, and especially in Capan-1 cells (Sauter et al., 2015). The upregulation was confirmed by an increase in TMEM16A protein expression for all three cell lines. Furthermore, it was shown that TMEM16A carries the major component of CaCC current in these cell lines (Sauter et al., 2015). Moreover, the authors found that knockdown of TMEM16A had no effect on proliferation. Inhibition, by T16A_inh_-A01 or other CaCC inhibitors, failed to affect PDAC cell lines proliferation, while T16A_inh_-A01 had a significant effect on the control cell line cell proliferation, which almost completely lack the expression of TMEM16A. These results suggest that the inhibition by T16A_inh_-A01 is unspecific for TMEM16A, and that this channel has no implication in proliferation, at least in these three PDAC cell lines (Sauter et al., 2015). According to the role of TMEM16A in migration, gene silencing reduced the migratory capability of AsPC-1 and BxPC-3 cells, where the inhibition with T16A_inh_-A01 was ineffective (Sauter et al., 2015). Other CaCC inhibitors caused a decrease in migration of BxPC-3 cells. Nevertheless, Capan-1 cells showed the highest expression of TMEM16A, the migration was very slow, suggesting that TMEM16A is not implicated in the role of migration in Capan-1 cells and supporting that TMEM16A has different roles in carcinogenesis of PDAC cells (Sauter et al., 2015).

Another recent study has performed a database investigation on the expression of TMEM16A and found that mRNA TMEM16A expression is upregulated in pancreatic cancer (Crottes et al., 2019). The authors found that extracellular application of EGF increased [Ca^2+^]_i_ and the outward-rectifying Cl^-^ current, which were both inhibited by different TMEM16A inhibitors. The regulation of Cl^-^ currents and the Ca^2+^ response were probably due to SOC entry. Furthermore, silencing of TMEM16A in AsPC-1 cells reduced migration even under EGF treatments, while EGF induced migration in the control cell line. This indicates that TMEM16A is involved in EGF-induced PDAC migration and progression, probably through Ca^2+^ signaling. In addition, this study investigated the possible role of TMEM16A to classify PDAC patients (Crottes et al., 2019). They found 10 genes involved in EGF-induced TMEM16A-dependent Ca^2+^ signaling, which could distinguish neuro-endocrine tumors from other pancreatic cancers. In PDAC, these genes formed three clusters with different genetic profiles that could reflect different molecular characterizations (Crottes et al., 2019).

Another TMEM16 protein expressed in pancreatic cancer is the TMEM16J protein, which also has been found to be overexpressed (Jun et al., 2017). TMEM16J is not a well characterized protein, but it is proposed that it might function as a cation channel activated by the cAMP/PKA signaling pathway (Falzone et al., 2018; Kim et al., 2018). An upregulation of TMEM16J gene-, mRNA-, and protein overexpression were found in AsPC-1, BxPC-3, and Capan-2 cell lines and a small overexpression in PANC-1 cells. An overexpression of TMEM16J in PANC-1 cells resulted in phosphorylated ERK1/2 levels, but not total ERK1/2 levels. Furthermore, both EGFR and phosphorylated EGFR levels were upregulated in PANC-1 cells overexpressing TMEM16J and an immunoprecipitation assay revealed that both TMEM16A and TMEM16J formed protein complexes with EGFR, but the binding affinity for TMEM16J was 132% higher, than for the one of TMEM16A (Jun et al., 2017), suggesting that TMEM16J are involved in upregulation and activation of EGFR. In contrary, a knockdown of TMEM16J in AsPC-1 cells resulted in inhibition of phosphorylated ERK1/2, EGFR and phosphorylated EGFR and a decreased proliferation rate. These results were confirmed *in-vivo*, were a xenograft mouse model was made by implanting PANC-1 cells stably overexpressing TMEM16J. It was shown that tumor growth was significantly increased and immunohistochemistry of these tumors confirmed the TMEM16J overexpression (Jun et al., 2017). These results indicate that TMEM16J is implicated in cell proliferation and tumor growth. Another member of the TMEM16 family, TMEM16E, has been shown to be implicated in PDAC. It is not yet clear whether the TMEM16E protein function as an ion channel or scramblase (Falzone et al., 2018). It has been shown, by immunohistochemical analysis, that TMEM16E is entirely expressed in PDAC but not in normal pancreatic tissue (Song et al., 2019). The highest expression of both mRNA and protein of TMEM16E was found in PANC-1 cells. The impact of TMEM16E on migration was investigated by a wound-scratch assay and a siRNA knockdown of TMEM16E showed a significant decrease in PANC-1 cell migration (Song et al., 2019). Even though, it should be mentioned that the authors do not account for the possible effect of proliferation in this assay. The migration was in some ways confirmed by the downregulation of vimentin protein expression, compared to the control, which showed a higher expression of vimentin, suggesting that TMEM16E is implicated in migration of PANC-1 cells. In addition, the proliferation of PANC-1 cells was significantly decreased upon knockdown of TMEM16E suggesting its role in proliferation (Song et al., 2019). This assay supports the speculation on the TMEM16E role in migration.

Besides being activated by Ca^2+^, CaCC can also be activated and regulated by specific proteins, namely Calcium-activated Chloride channel regulators (CLCAs) also called Calcium Chloride channel accessory proteins. CLCAs are expressed in different types of cancer and have been implicated in regulation of proliferation, migration and metastasis (Yurtsever et al., 2012; Lang and Stournaras, 2014; Stock and Schwab, 2015). CLCA1 has been shown to be overexpressed in pancreatic cancer (Hu et al., 2018a; Hu et al., 2018b). However, the expression pattern and underlying molecular mechanism of its role in PDAC is less known. Finally, low gene expression of Chloride Channel Kb (*CLCNKB*) and Chloride Voltage-Gated Channel 1 (*CLCN1)* have been reported in human PDAC tissue compared to normal pancreatic epithelium (Zaccagnino et al., 2016).

#### CFTR

It has been shown that CFTR is expressed in some PDAC cell lines. An early study showed that CFTR only was expressed in Capan-1 cells among nine different pancreatic cell lines and that the expression varied as a function of confluence (Chambers and Harris, 1993). Singh et al., confirmed the almost non-existent expression of CFTR in PDAC cell lines. Indeed, mRNA levels were detectable in normal pancreatic tissue and three (Capan-1, Suit2 and SW1990) out of 16 pancreatic cell lines (Singh et al., 2007). Furthermore, Zaccagnino et al., reported the downregulation of CFTR, at gene-level, in human PDAC tissue compared to normal pancreatic epithelium. This downregulation was associated with gene expression of EMT transcription factors (Zaccagnino et al., 2016). Furthermore, Singh et al., showed that wild type CFTR negatively regulated MUC4 expression while silencing of CFTR upregulated MUC4 expression. As MUC4 is a protein involved in tumor migration and metastasis, the negative regulation by CFTR indicates a protective role and a tumor suppressing function by inhibiting MUC4 and hence pancreatic cancer progression (Singh et al., 2007).

A recent study has investigated CFTR expression in patient derivated PDAC organoids, in order to enable routine organoid subtyping for personalized treatment (Hennig et al., 2019). It has been suggested that subtyping could be based on the expression of cytokeratin 81 (KRT81) and hepatocyte nuclear factor 1A (HNF1A). As the antibody for HNF1A was no longer available, the authors permitted CFTR to replace it as a potential marker instead of HNF1A (Hennig et al., 2019). Organoids can be categorized into the established quasi-mesenchymal, exocrine-like, and classical subtypes. Immunofluorescence staining showed a mutual expression pattern where exocrine-like organoids were CFTR^+^/KRT81^-^ and quasi-mesenchymal CFTR^-^/KRT81^+^. The protein expression revealed by IF was compared to mRNA levels of CFTR, which matched in 8 out of 10 cases (Hennig et al., 2019). In addition, it was confirmed, by immunohistochemical analysis, that both CFTR and KRT81 were preserved in 6 out of 7 tumors, indicating that the organoids had the same subtype as their primary tumor (Hennig et al., 2019). These results suggest that CFTR could be a supplement marker for HFN1A and that CFTR/KRT81 together might be a suitable way to evaluate subtype organoids for personal treatments (Hennig et al., 2019).

#### Cl^-^ Intracellular Channel Proteins (CLICs)

CLICs are ubiquitously expressed and have been identified in several types of cancer, where they are either overexpressed or downregulated compared to the normal tissue (Peretti et al., 2015). In PDAC, CLICs are mostly found upregulated, even though their specific role in PDAC progression and development is not yet understood. CLIC2, CLIC3, and CLIC5 have been shown to be expressed at mRNA and protein levels in a HPAF cell line. By an electrophysiological study, the authors revealed that there was no single channel/conductance for apical Cl^-^ secretion, but that these CLICs rather contributed to provide a constant net conductance across the plasma membrane (Fong et al., 2003). Another study has shown the importance of CLIC3 in PDAC, as immunohistochemical analysis and mRNA levels showed an overexpression of CLIC3 in PDAC tissue compared to normal pancreatic tissue (Dozynkiewicz et al., 2012). It was also found that CLIC3 in collaboration with Rab25 promoted cancer cell invasion and migration by integrin recycling from late endosomes/lysosomes (Dozynkiewicz et al., 2012). CLIC1 was overexpressed in primary tumors compared to normal pancreatic tissue, and strongly expressed in MiaPaCa-2 and PANC-1 cells (Lu et al., 2015). Silencing of CLIC1 showed a significant decrease in the proliferation rate, colony formation and the invasive abilities of both MiaPaCa-2 and PANC-1 cells, suggesting that CLIC1 contributes to the aggressive role of these PDAC cells (Lu et al., 2015). In addition, gene expression levels of CLIC5 has been found to be downregulated in PDAC tissue, compared to normal pancreatic epithelium and to be associated with gene expression of transcription factors related to cell differentiation (Zaccagnino et al., 2016).

### Aquaporins (AQPs) in PDAC

AQPs are expressed in various types of cancers and are predicted to be key regulators in tumor development and progression (Papadopoulos and Saadoun, 2015). The expression and role of AQPs in PDAC is poorly studied, yet few studies have described their involvement in PDAC progression (Burghardt et al., 2003; Direito et al., 2017; Huang et al., 2017; Zou et al., 2019). Burghardt and co-workers have found mRNA expression of AQP1, AQP3, AQP4, AQP5, and AQP8 in PDAC. All subtypes were expressed in solid tumors, where only AQP3, AQP4, and AQP5 were expressed in PDAC cell lines (Burghardt et al., 2003). Further studies have found an upregulation of AQP1 and AQP3 in PDAC tissue compared to normal pancreatic tissue (Zou et al., 2019). The expression of AQP1 correlated with the expression of AQP3, suggesting that these two channels cooperate during PDAC development. Another study has shown the overexpression of AQP3 and AQP5 in PDAC tissue (Direito et al., 2017). AQP5 localization in PDAC was found to be in the entire plasma membrane and in the cytoplasm of ducts cells, where in normal pancreas the localization is in the apical membrane. Furthermore, AQP5 and AQP3 were suggested to be involved in proliferation and tumor transformation as a simultaneous overexpression was found to be correlated with an increased expression of EGFR, Ki-67, CK7, and a decrease of E-cadherin and increase of Vimentin (Direito et al., 2017). Another study investigating AQP has, through TCGA analysis, revealed that AQP3 shows the highest expression among AQPs in PDAC (Huang et al., 2017). The authors investigated the role of AQP3 further, with a focus on how microRNA (miR-874) regulates gene expression and post-translational events in PDAC. In a panel of eight pancreatic cell lines, they detected that cell lines with high AQP3 mRNA levels had lower miR-874 levels, where cell lines with high miR-874 had lower AQP3 levels suggesting that AQP3 expression is regulated by miR-874 (Huang et al., 2017). It was found that both modulation of AQP3 and miR-874 altered the expression and activity of mTOR and its downstream target S6, suggesting that an overexpression of AQP3 is associated with proliferation and cell survival by mTOR signaling in PDAC. In contrast to other studies, Zaccagnino et al. (2016) showed a downregulation of AQP3 and AQP8 expression in PDAC tissue compared to normal pancreatic one. Furthermore, they showed that AQP3 expression was associated with several cell differentiation related transcription factors.

### Sodium Channels in PDAC

#### ASIC

It has recently been shown that acid-sensing ion channels (ASICs), an H^+^-gated subgroup of ENaC, are expressed in PDAC cell lines and tissue (Zhu et al., 2017). ASIC1 and ASIC3 were found functionally expressed and mRNA and protein expression were also found in PDAC cell lines. In all cases, the expression was upregulated compared to the normal control cell line. These results were confirmed in PDAC tissue where immunohistochemical analysis and qPCR revealed the overexpression compared to non-cancerous pancreatic tissue, suggesting that ASIC1 and ASIC3 have a pathophysiological role in PDAC (Zhu et al., 2017). Separate inhibition or knockdown of ASIC1 and ASIC3 decreased the acidity-promoted invasion and migration capacity of PDAC cell lines, but did not decrease the proliferation rate, suggesting that ASIC1 and ASIC3 are involved in the metastatic process of PDAC, but not tumor cell growth (Zhu et al., 2017). Furthermore, it was shown that ASIC1 and ASIC3 are involved in acidity-promoted EMT, as silencing or inhibition of ASIC1 or ASIC3 in PDAC cells showed decreased protein expression of mesenchymal markers Vimentin, N-cadherin, Snail, and ZEB1, while the epithelial marker E-cadherin showed increased protein expression. In contrary, PDAC cells overexpressing ASIC1 and ASIC3 showed an increase in mesenchymal markers and a decrease in epithelial markers, under acidic conditions. This was confirmed in human PDAC tissue samples by IF analysis. It was further investigated whether this mechanism was regulated by [Ca^2+^]_i_, where it was found that inhibition of ASIC1 or ASIC3 resulted in a decrease of [Ca^2+^]_i_ upon acidification. In addition, the removal of [Ca^2+^]_i_ upon acidic conditions decreased mesenchymal markers and increased the epithelial ones. It was determined that the RhoA pathway, which is involved in cytoskeleton re-arrangement and cell migration, was a major effector of EMT induced by ASIC1/3-[Ca^2+^]_i_ activation in acidic conditions (Zhu et al., 2017). The role of ASIC1 and ASIC3 was further confirmed *in-vivo*, where a xenograft mouse model injected with BxPC-3 cells with a stable knockdown of ASIC1 and ASIC3 showed a significant decrease in lung and liver metastasis, but no obvious effect on tumor growth (Zhu et al., 2017).

#### VGSCs

Another subfamily of Na^+^ channels, namely voltage gated sodium channels (VGSCs), has shown to be implicated in cancer progression (Angus and Ruben, 2019). An early study has shown that Ca^2+^ blockers Phenytoin and Verapamil inhibited the growth of pancreatic cancer cell lines MiaPaCa-2 and CAV, both *in-vitro* and *in-vivo* (Sato et al., 1994). Phenytoin and Verapamil were chosen because they appeared to be blocking different Ca^2+^ channels; T-type and L-type voltage dependent Ca^2+^ channels, respectively (Sato et al., 1994). It has been suggested that this growth-inhibition of pancreatic cancer cell was rather due to the block of VGSC than the block of Ca^2+^ channels, as both Phenytoin and Verapamil show high affinity for VGSC in the inactivated state of the channel (Ragsdale et al., 1991; Koltai, 2015). In addition, the expression of VGSC (*SCN9A* and *SCN3A*) was downregulated in PDAC (Zaccagnino et al., 2016).

## Ionotropic Receptors in PDAC

### Purinergic Receptors (P2XR) and N-Methyl-D-Aspartate Receptors (NMDAR)

Different types of ionotropic receptors including P2XR and NMDAR have been reported to be expressed in PDAC (Kunzli et al., 2007; Hansen et al., 2008; Burnstock and Novak, 2012; Li and Hanahan, 2013; North et al., 2017). Among P2XR, P2X7R is the most well described (Kunzli et al., 2007; Hansen et al., 2008; Burnstock and Novak, 2012).This ionotropic receptor has shown to be overexpressed in PDAC cell lines and tissue (Kunzli et al., 2007; Giannuzzo et al., 2015), and to be implicated in the proliferating, apoptotic, migrating, and invading processes of PDAC (Kunzli et al., 2007; Hansen et al., 2008; Giannuzzo et al., 2015; Giannuzzo et al., 2016; Choi et al., 2018). In addition, the expression of NMDAR was found in both PDAC cell lines and PDAC tumors, and their inhibition and blocking resulted in reduced different PDAC cell lines viability and survival (Li and Hanahan, 2013; North et al., 2017). Furthermore, an inhibition of NMDAR prevented growth of tumor xenografts (Li and Hanahan, 2013; North et al., 2017).

## Ion Channels as PDAC Biomarkers

A growing number of studies have investigated ion channel expression in pancreatic cell lines and human tissues, showing modulation of mRNA and/or protein expression between normal and cancer cells. Among all the studied channels, only CFTR has lower expression in cancer cell lines compared to normal cells (Singh et al., 2007), while Kv1.3, Kv7.1, and TASK-1 were downregulated in PDAC tissue compared to healthy tissue (Brevet et al., 2009; Williams et al., 2013; Tawfik et al., 2020), suggesting a protective role and tumor suppressive function for these channels. Although most of the ion channels are overexpressed in PDAC, studies on ion channel expression patterns in correlation with clinical parameters are more limited.

### Diagnostic Markers

Some attention has been given to the connection between pancreatic cancer risk and CFTR deficiency. Mutations in the *CFTR* gene cause the hereditary life shortening disease cystic fibrosis (CF). Severe clinical manifestations occur upon CF in secretory epithelial tissues and in pancreas, mutations causing loss of function lead to pancreatic insufficiency (Wilschanski and Novak, 2013; Castellani and Assael, 2017). Different cohort studies have investigated how different variants of *CFTR* affect the risk of pancreatic cancer (Sheldon et al., 1993; Neglia et al., 1995). It has been shown that CF patients present an elevated risk to develop pancreatic cancer, even though the overall risk of developing cancer is the same as for the general population (Sheldon et al., 1993; Neglia et al., 1995). Furthermore, studies also indicate that patients who are *CFTR* mutant carriers develop pancreatic cancer in a younger age, compared to patients carrying a wildtype form of *CFTR* (McWilliams et al., 2010; Hamoir et al., 2013), and patients carrying a germline mutation to some degree have an increased risk of developing PDAC (Cazacu et al., 2018). One mechanism of which a *CFTR* mutation could cause pancreatic cancer is by the defect of CFTR and ion transport leading to dysregulated mucus secretion and obstruction of the pancreatic ducts, which all are events that could result in pancreatitis (McWilliams et al., 2010). Patients with chronic pancreatitis have a 26-fold higher risk for developing pancreatic cancer compared to the general population (Lowenfels et al., 1993; Kirkegard et al., 2017), suggesting that *CFTR* mutation could be considered as a new risk factor for developing PDAC.

In order to discriminate pancreatic premalignant/malignant lesions from benign lesions, an explorative proteomic approach was performed on a cohort of 24 patients using targeting mass spectrometry analysis of different biomarkers (Jabbar et al., 2018). This study proposed CLCA1 to be a supportive marker, which together with mucin-5AC (MUC5AC) and prostate stem-cell antigen (PSCA) could distinguish cystic precursor lesions from PDAC, suggesting that CLCA1 is a potential biomarker in PDAC diagnosis.

### Prognostic Markers of Cancer Progression and Aggressiveness

Different studies have investigated ion channels as potential biomarkers of PDAC development and progression. Using immunohistochemical analysis, high KCa3.1 expression in PDAC tissue was correlated with TNM stages III and IV (Jiang et al., 2017), and high expression of STIM1 was correlated with tumor grade (Wang et al., 2019). Upregulation of Kv11.1 expression was associated with advanced tumor grade and high expression of Ki67 proliferative marker (Lastraioli et al., 2015b), whereas TRPV6, TRPM8 and AQP1/AQP3 channels were positively correlated with tumor stages III and IV and large tumor size (Yee et al., 2014; Du et al., 2018; Liu et al., 2018; Song et al., 2018; Zou et al., 2019). Finally, TRPM7 and Cl^-^ intracellular channel proteins (CLIC1-3). CLIC1 overexpression was shown to be correlated with the three clinical parameters: advanced tumor grade, advanced tumor stage and large tumor size (Rybarczyk et al., 2012; Lu et al., 2015; Yee et al., 2015; Jia et al., 2016). These results suggest that all the ion channels cited above are associated with pancreatic tumor growth.

Regarding the metastatic status, immunohistochemistry experiments showed that TRPV6 expression was higher in cases where PDAC was infiltrating (Song et al., 2018). The same results were observed at mRNA and protein levels for CLIC3, with a highly detectable expression in regions where the tumor was invading normal pancreatic tissue (Dozynkiewicz et al., 2012). Other studies revealed higher TRPM7 and TRPM8 staining in metastatic tumors than in non-metastatic tumors (Yee et al., 2015; Liu et al., 2018), which was confirmed by qPCR for TRPM8 (Du et al., 2018). AQP1 and AQP3 were also more expressed in PDAC patients with lymph node metastasis and invasion, than in non-invasive cancers (Zou et al., 2019), whereas overexpression of Kir3.1 potassium channel (GIRK1) was not found to be correlated with metastatic status (Brevet et al., 2009).

Gene expression correlation analysis demonstrated that TRPM2 is strongly correlated with different genes including toll-like receptor 7 (TLR7) (Lin et al., 2018), which has already been associated with PDAC progression (Ochi et al., 2012; Grimmig et al., 2015; Wang et al., 2016). Moreover, AQP1 and AQP3 protein expression was highest in poorly differentiated tumors (Direito et al., 2017; Zou et al., 2019), whereas AQP5 is more expressed in moderately differentiated tumors (Direito et al., 2017), suggesting that AQPs are associated with tumor aggressiveness.

Finally, most of the studied ion channels in PDAC tissue were associated with overall survival of the patients. The authors usually used immunohistochemical staining on large cohorts and Kaplan-Meier survival analysis, to observe a correlation between high channel expression and short patient survival. This is the case for KCa3.1 (Jiang et al., 2017), STIM1 (Wang et al., 2019), TRPM8 (Liu et al., 2018), TRPV6 (Song et al., 2018), TMEM16J (Jun et al., 2017), CLIC1 (Lu et al., 2015; Jia et al., 2016), and AQP1/AQP3 (Zou et al., 2019). The same correlation was obtained on gene expression using qPCR for TRPM8 (Du et al., 2018) or TCGA for TRPM2 (Lin et al., 2018) and TMEM16A (Crottes et al., 2019). Furthermore, the mutation status of TRPM2 was also analyzed using Kaplan-Meier in 10 patients out of 159, and the mutated *TRPM2* gene revealed a negative correlation with patient survival, compared to patients expressing wildtype *TRPM2* (Lin et al., 2018). Studies on shorter cohorts also revealed that high protein expression of Kv11.1 and TRPM7 channels are inversely correlated with overall survival using Pearson correlation on 18 patients, and multivariate overall survival analysis on 44 samples, respectively (Rybarczyk et al., 2012; Lastraioli et al., 2015a) Comparison of ion channels expression level in 9 patients with short survival (<12 months) and 10 patients with long survival (>45 months) showed that short survival was correlated with high expression of CLIC3 and low expression of CLCA1 (Hu et al., 2018b). This low CLCA1 expression correlated with shorter disease-free survival was confirmed using tissue microarrays, immunohistochemistry and Kaplan-Meier analysis in 140 patients (Hu et al., 2018a). Except for CLCA1 which could be proposed as a good prognostic marker, all the other studied ion channels could be proposed as poor prognostic markers.

These studies on human tissues highlighted the major clinical relevance of ion channels expression in pancreatic cancer development (**Table 2** and **Figure 3**). Indeed, the expression of potassium, calcium, chloride channels, and aquaporins is mainly associated with aggressiveness and invasiveness and inversely correlated to patient survival, suggesting that they may be potential markers of poor prognosis.

### Therapeutic Targets

In general, PDAC cells are resistant to pro-apoptotic reagents, and overexpression of ion channels was shown to be involved in this resistance. Knockdown of TRPM7 in combination with gemcitabine treatment enhanced cytotoxicity in PANC-1 cells even though the precise mechanisms are not yet determined (Yee et al., 2012b), whereas the silencing of TRPV6 in Capan-2 PDAC cells resulted in a significant increase of sensitivity to the chemotherapeutic reagent oxaliplatin, but had little effect on gemcitabine and cisplatin treatments (Song et al., 2018). Another study has shown that silencing of TRPM8 in combination with gemcitabine suppressed the proliferation and invasion properties of PANC-1 and BxPC-3 cells. In addition, gemcitabine-sensitivity depended on TRPM8 silencing in these cell lines, where mRNA level of multi-drug related proteins was decreased, and expression of apoptosis-related proteins was also affected, suggesting that TRPM8 is involved in multi-drug resistance and apoptosis of PDAC cells (Liu et al., 2018). PANC-1 cells apoptosis was also increased after treatment with chemotherapeutic reagents 5-fluorouracil or gemcitabine in combination with a knockdown of ORAI1, STIM1, or both (Kondratska et al., 2014). Furthermore, it was shown that cells treated with either 5-fluorouracil or gemcitabine increased ORAI1 and STIM1 expression as well as SOC entry suggesting that ORAI1 and STIM1 confer resistance to chemotherapy, probably through the increase of SOC entry (Kondratska et al., 2014). More recently, STIM1 was found to be involved in gemcitabine resistance in PDAC (Zhou et al., 2020). The transcriptome sequencing analysis in established gemcitabine resistant PDAC cell lines, showed that STIM1 was significantly upregulated in the gemcitabine resistant cell lines, compared to the parental cell line (Zhou et al., 2020). Among the chloride channels, knockdown of TMEM16J provided an additive effect on inhibiting proliferation upon treatment with gemcitabine and erlotinib, suggesting that a TMEM16J inhibitor can help to prevent gemcitabine resistance associated with the prolonged use of gemcitabine (Jun et al., 2017). The team of Arcangeli has been investigating another therapeutic perspective with the development of a novel anti-Kv11.1 antibody-conjugated PEG-TiO_2_ nanoparticles for targeting PDAC cells (Sette et al., 2013).

## Conclusion

Increasing evidence indicates that ion channels are involved in the regulation of cancer proliferation, apoptosis, chemo-resistance, migration, and invasion. The field of ion channels in PDAC still constitutes a novel area of research and even studies conclude their involvement in the malignancy and aggressiveness of PDAC, only relatively few studies provide the complete signaling pathways. Moreover, the majority of the studies cited in this review were carried out on 2D cultured cell lines. It appears thus necessary to develop and/or increase better approaches (organoids, 3D culture, and/or animal models) to investigate the candidate channel(s) as well as its (their) function and associated signaling pathways in PDAC. However, recently, an increasing number of publications on signaling in pancreatic cancer take the tumor microenvironment into-account. This reflects the interest in ionic channels and their potential promising use as therapeutic targets in the fight against pancreatic cancer.

## Author Contributions

JS, ID-D, AA, and HO-A: design and manuscript preparation. All authors contributed to the article and approved the submitted version.

## Funding

JS is grateful for the funding by the Marie Skłodowska-Curie Innovative Training Network (ITN) Grant Agreement number: 813834 - pHioniC - H2020-MSCA-ITN-2018. HO-A is grateful for the funding by the Ministère de l’Enseignement Supérieur et de la Recherche, the Région Hauts-de-France (Picardie), the FEDER (Fonds Européen de Développement Économique Régional), the Université Picardie Jules Verne, and the Ligue Contre le Cancer (Septentrion).

## Conflict of Interest

The authors declare that the research was conducted in the absence of any commercial or financial relationships that could be construed as a potential conflict of interest.
